# Adaptive dimensionality reduction for neural network-based online principal component analysis

**DOI:** 10.1371/journal.pone.0248896

**Published:** 2021-03-30

**Authors:** Nico Migenda, Ralf Möller, Wolfram Schenck

**Affiliations:** 1 Center for Applied Data Science Gütersloh, Faculty of Engineering and Mathematics, Bielefeld University of Applied Sciences, Bielefeld, Germany; 2 Computer Engineering Group, Faculty of Technology, Bielefeld University, Bielefeld, Germany; Fuzhou University, CHINA

## Abstract

“Principal Component Analysis” (PCA) is an established linear technique for dimensionality reduction. It performs an orthonormal transformation to replace possibly correlated variables with a smaller set of linearly independent variables, the so-called principal components, which capture a large portion of the data variance. The problem of finding the optimal number of principal components has been widely studied for offline PCA. However, when working with streaming data, the optimal number changes continuously. This requires to update both the principal components and the dimensionality in every timestep. While the continuous update of the principal components is widely studied, the available algorithms for dimensionality adjustment are limited to an increment of one in neural network-based and incremental PCA. Therefore, existing approaches cannot account for abrupt changes in the presented data. The contribution of this work is to enable in neural network-based PCA the continuous dimensionality adjustment by an arbitrary number without the necessity to learn all principal components. A novel algorithm is presented that utilizes several PCA characteristics to adaptivly update the optimal number of principal components for neural network-based PCA. A precise estimation of the required dimensionality reduces the computational effort while ensuring that the desired amount of variance is kept. The computational complexity of the proposed algorithm is investigated and it is benchmarked in an experimental study against other neural network-based and incremental PCA approaches where it produces highly competitive results.

## Introduction

Data is streaming from all areas of our live. The data streams are often available in large quantities and have a high dimensionality. Applying machine learning algorithms to high-dimensional data streams is a challenging task. This task requires the use of efficiently applicable online machine learning algorithms and memory storage [1]. A few algorithms, e.g. kernel methods [2, 3], are well suited to work under these conditions. However, working in a high-dimensional space comes at a price in form of an increased prediction error, impaired interpretability and higher computational costs [4]. In order to solve this problem, online dimensionality reduction is necessary so that a larger variety of machine learning algorithms can be applied to the lower-dimensional stream of data. Dimensionality reduction is the task of transforming high-dimensional data into a lower-dimensional representation [5]. The reduced representation has ideally a dimensionality close to the intrinsic dimensionality of the data stream. The intrinsic dimensionality describes how many variables are needed to generate a good approximation. In that way, online dimensionality reduction methods mitigate the curse of dimensionality, reduce the computational effort of machine learning algorithms, and facilitate the visualization of high-dimensional data streams.

### Problem statement

Determining the optimal number of dimensions, e.g. principal components in PCA, is routinely applied for offline dimensionality reduction, but not for online dimensionality reduction with streaming data. Streaming data is possibly subject to noise, drift or other influences, so that the optimal dimensionality has to be adjusted continuously in order to maintain the desired amount of variance in PCA. Therefore, for an online method to be effective, it is necessary to continuously add or remove dimensions with each data point when appropriate [6]. Existing methods in neural network-based PCA [7] and incremental PCA [8, 9] are limited to an increment of one and are therefore unable to account for abrupt changes in data variance. When the dimensionality is too small, the quality of the reconstructed signal suffers. On the other hand, training many unnecessary components increases the computational effort. Therefore, the efficient adjustment of dimensionality by an arbitrary number after the presentation of each data point is necessary.

### Objectives and structure

The contribution of this work is the continuous dimensionality adjustment in neural network-based PCA by arbitrary steps, without the constraint to learn all principal components at every timestep. Therefore, stopping rules previously not directly applicable to neural network-based PCA are extended for online learning. Being able to adjust the dimensionality by more than one at every data point presentation, lets the PCA respond faster to changes in data variance. This leads to a better data representation. To achieve this, a novel algorithm that exploits natural characteristics of neural network-based PCA is proposed. The method is an extension to neural network-based PCA.

In this paper, a comprehensive experimental study is carried out. The goal is to demonstrate that the proposed online algorithm determines the correct number of meaningful principal components long before all data points are presented. In order to rate the quality appropriately, the chosen (freely available) data sets vary in their characteristics over a wide range.

The paper is organized as follows: First, an overview of state of the art algorithms for dimensionality reduction on data streams and in particular neural network-based and incremental PCA is given. Then it is shown why the stopping rules for offline PCA are not directly applicable to neural network-based PCA. As a consequence, a novel approach to solve this problem is presented. A comprehensive experimental study is carried out that shows the successful application to a variety of data sets. Furthermore, different versions of the proposed algorithm are benchmarked against each other. Afterwards, the improved robustness and adaptation speed is demonstrated by benchmarking the proposed algorithm against (1) an alternative neural network-based and (2) an incremental PCA approach. Lastly, conclusions are given in the final section.

## State of the art

Traditional methods for dimensionality reduction are of linear nature [10]. These methods linearly map high-dimensional data into a lower-dimensional form. They are capable of preserving a wide range of data features of interest, e.g. covariance, correlation between data sets and the input-output relationship. The most used family of linear dimensionality reduction techniques is based on orthogonal projections [10]. These methods are popular because of their simple geometrical interpretation and the low-dimensional view of high-dimensional data.

One orthogonal projection method is “Principal Component Analysis” (PCA) [11, 12]. PCA is a method that transforms the data by projecting it onto a set of orthogonal axes. Removing the second-order dependencies yields an orthonormal basis the directions of which are uncorrelated. PCA is suitable when the dimensions in the original data space are related to each other so that it is possible to describe the relationships using fewer dimensions than are actually present. While PCA is maximizing the variance of the projected data, another objective is to maximize the scatter of the projections. This approach is pursued in “Multidimensional Scaling” (MDS) [13] under the expectation that maximizing the scatter yields the most informative data projection. While the mapping is not necessarily linear, it is commonly assumed. The linear techniques for dimensionality reduction described above use an orthogonal mapping, while other methods simplify further to an unconstrained optimization. “Independent Component Analysis” (ICA) [14] belongs to this family of linear dimensionality reduction methods. ICA specifies the data as a mixture of unknown and independent sources. It finds the demixing matrix so that the independent sources are recovered. In cases where the full set of dimensions is preserved, no dimensionality reduction is performed. The case of interest is therefore the undercomplete ICA [15, 16].

All presented and many more [10] linear methods score through their simplicity and have efficient online applicable versions [17, 18], enabling dimensionality reduction on a data stream. In addition, they have fast computation times, have only a few hyper-parameters to tune and are easy to interpret. When it comes to describing non-linear data sufficiently, linear approaches for dimensionality reduction can be combined with local methods, e.g. clustering, to describe non-linear data with local linear models [19, 20]. In this way the dimensionality of each subspace can be adjusted individually, resulting in an improved representation of the data.

However, when dealing with highly non-linear data, non-linear methods for dimensionality reduction are often chosen [21]. The method with the greatest surge in use for non-linear dimensionality reduction is the “Autoencoder” (AE) [22]. AE are unsupervised artificial neural networks that first compress (encode) the data into a low-dimensional subspace and then reconstruct (decode) the data back into the original space. This method is online applicable and well able to represent complex non-linear data with a low prediction error. On the downside, it is costly to update non-linear methods continuously and they have many hyperparameters to tune. Therefore, linear techniques are preferable for many applications, and the focus of this work lies on further improving linear methods, in particular in a streaming setting in which the subspace is updated without knowledge of the data history [23].

The foundation of incremental PCA and incremental SVD are classical numerical methods in which the set of eigenvalues and eigenvectors is updated incrementally [8, 24]. The learning paradigm is based on solving the intermediate eigenproblem repeatably for every training sample [25]. By observing sample by sample and not the entire data set at once, both the memory usage and computational complexity are reduced [26]. Most approaches are free of directly recalculating the covariance matrix, further reducing the computational complexity. Still, most methods assume a fixed mean when updating the eigenvalues and eigenvectors or that the data is inherently zero-mean. Only in works based on [8, 27] the change of mean is considered. Therefore, it is unnecessary to accumulate data before performing an update and an updated mean is always available to incorporate new data. In addition, at each data point presentation, a model with a dimensionality increased by one is estimated [8], and a forgetting factor may be used [26, 27] to either keep or discard past observations.

Online PCA algorithms which rely on principles from neural computation, also referred to as neural network-based PCA, are an efficient approach to update the principal components after each presentation of a data point. A variety of neural network-based PCA learning algorithms were proposed of which the Hebbian and Oja’s learning rules [25, 28, 29] are the foundation. In these rules, each principal component corresponds to a neuron and is defined by the input weights of the corresponding neuron. The Hebbian learning rule is biologically inspired so that synaptic weights adapt in proportion to the correlation between the presynaptic and postsynaptic signals. To prevent divergence during the training process, the weights are normalized to unity with each presentation of data. This normalized form of the Hebbian rule [30] is the basis for many other algorithms, such as Oja’s rule. In Oja’s learning rule, a weight decay term is added to the Hebbian rule for stabilization. PCA algorithms such as Hebbian rule-based algorithms can be derived by optimizing an objective function using the gradient-descent method. Both the Hebbian and Oja’s algorithm are sensitive to their hyperparameters, e.g. learning rate, so that tuning these parameters to achieve a fast convergence speed while maintaining stability is difficult. To overcome this drawback, approaches based on recursive least squares (RLS) have been suggested [31]. All RLS-based PCA algorithms exhibit fast convergence, stability and high tracking accuracy, and are suitable for slowly varying non-stationary vector stochastic processes. This approach was further extended towards robust recursive least squares (RRLS) algorithms in which increasing the number of neurons does not affect the previously extracted principal components [32]. The Hebbian and Oja rules are closely related to the RRLS-algorithm by a suitable selection of the learning rates. According to [32], the RRLS-algorithm provides the best performance in terms of convergence speed as well as steady-state error. The neural network-based PCA algorithms mentioned so far do not include the eigenvalue estimation in the update equations of the weights. In coupled learning rules for neural network-based PCA, eigenvalues and eigenvectors are simultaneously estimated [33]. This approach solves the speed/stability problem that exists in non-coupled PCA, so that the speed is the same in all directions and mainly depends on the principal eigenvalues of the covariance matrix. The online neural network-based PCA applied in this work is a RRLS-algorithm [34] with an adaptive learning rate control in which the eigenvalues and eigenvectors are trained based on their value in a descending order (S1 Appendix). In the following, the terms neural network-based PCA and online PCA are used synonymously for the sake of simplicity.

## Materials and methods

In the context of data analysis with big data [35, 36], dimensionality reduction methods [37, 38] are typically applied to reduce the set of attributes while preserving important data features. One of the approaches most used for dimensionality reduction is “Principal Component Analysis” (PCA).

### PCA in an offline or batch setting

**Fig 1:**
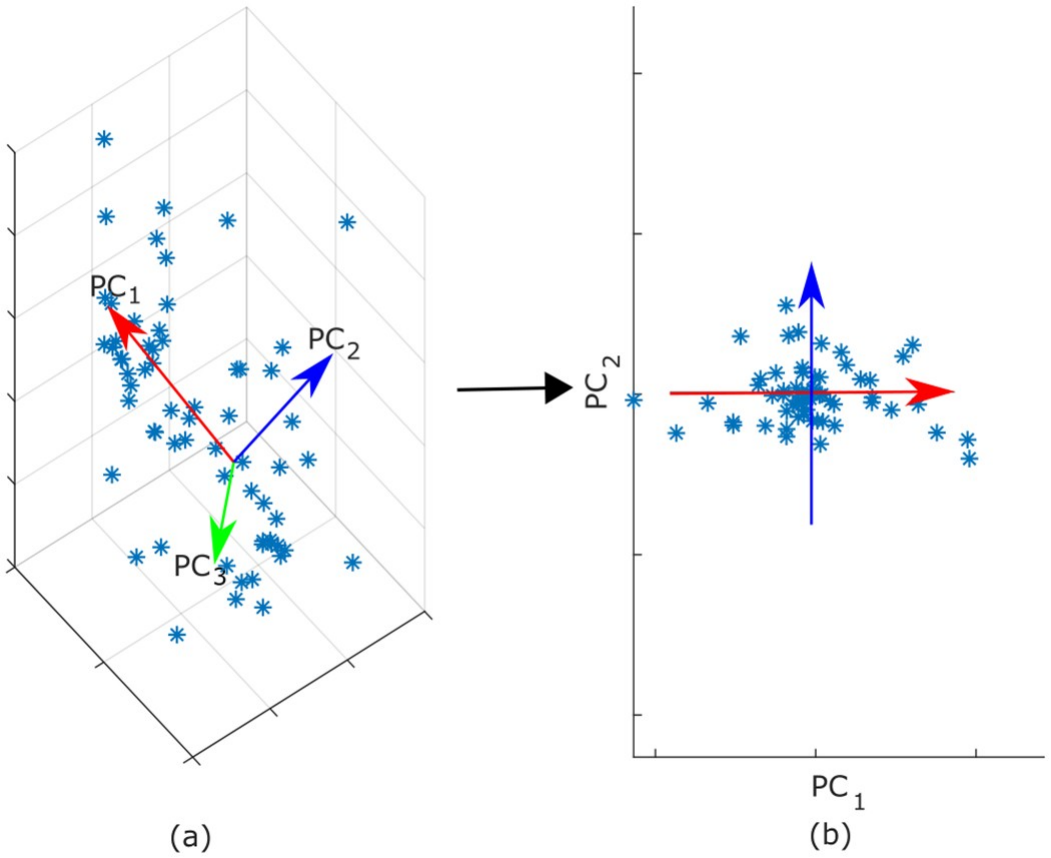
Dimensionality reduction process performed with a PCA: (a) Data distribution in a three-dimensional input space and the corresponding principal components; (b) Projection of the data into the two-dimensional space with the PC_1_, PC_2_ axes.

The basic idea of PCA is to preserve maximal variance for a data set with a minimal set of linear descriptors. High-dimensional data sets are projected onto a smaller number of dimensions maximizing the variance on the new axes. These components are orthogonal to each other. Fig 1a) shows an example of a three-dimensional distribution. The PCA would determine the three axes of the distribution in descending order of the variances of the data projections. The first principal component is the linear descriptor which represents the largest proportion of the overall data variance. With the value of the first principal component, the following principal components will represent the most additional variance. In the given example it might be sufficient to represent a data point by using only the value of the first two principal components (Fig 1b). Hence, an application of PCA is dimensionality reduction.

Classical offline PCA or batch-PCA [11, 12] applies an orthonormal transformation to transform a possibly correlated set of data into a set of linearly independent variables. High-dimensional pattern with *n* dimensions can be approximated by a lower-dimensional subspace of *m* dimensions IR^n^ → IR^m^. A PCA model describes the subspace with *m* principal components (with *m* ≤ *n*). In the following, the *n* × *m* matrix **W** denotes the estimated normalized eigenvectors **w**_*i*_, *i* = 1, …, *m* of the data covariance matrix, with one vector per column. These eigenvectors are identical to the principal components. The variance of the projection of the data distribution on the i^th^ principal component **w**_*i*_ is equal to the eigenvalue λ_*i*_. All eigenvalues λ_*i*_ are stored in a diagonal matrix **Λ** with a size of *m* × *m* in descending order. An estimation of the remaining eigenvalues in the *n* − *m* minor eigendirections can be derived from the residual variance *σ*^2^ [39, p. 93]. Additionally, a center vector **c** ∈ IR^n^ is required to center the PCA. This allows to represent multivariate data only with the matrices **W**, **Λ**, the vector **c** and *σ*^2^.

### PCA in a streaming setting

A streaming setting in PCA is characterized by sequentially arriving data points over a period of time during which the parameters describing the subspace are repeatedly updated. Over a period of time, the covariance matrix or the subspace can vary, so that tracking and reacting to such changes is necessary to maintain a best possible approximation. PCA algorithms capable of updating its set of parameters continuously without knowledge of the history of data are referred to as online PCA. Popular types of algorithms that fall under the term of online PCA are: incremental PCA [8] or incremental SVD [40] and neural network-based PCA [41]; the focus of this work lies on the latter.

#### Neural network-based PCA

Neural network-based PCA [25, 28, 42, 43] refers to typically unsupervised methods that estimate the eigenvalues λ_*i*_ and eigenvectors **w**_*i*_ online from the input data stream $\text{x}\in {\mathbb{R}}^{n}$. These methods are particularly useful for high-dimensional data streams since they avoid the computation of the large covariance matrix. In addition, they can track non-stationary data (i.e. data with a slowly changing covariance matrix). While the development of neural network-based PCA is described in the previous section, it is the focus of this section to provide a more technical view of the neural network-based PCA that is extended and benchmarked in this work [34]. The PCA extended in this work by an adaptive dimensionality adjustment is based on a robust recursive least square algorithm (RRLSA) [32] with interlocking of learning and Gram-Schmidt orthonormalization [34]. In this method, the eigenvectors are updated in a hierarchically way: The eigenvector with the largest eigenvalue is obtained using a single-unit learning rule applied to the original data. In order to obtain the next eigenvector corresponding to the second largest eigenvalue, the projection of the first eigenvector is subtracted from the data, so that a second single-unit network can be trained with these deflated vectors. Repeating this procedure yields up to *n* eigenvectors. However, the procedure can be stopped at any desired number. The update of each eigenvector **w**_*i*_ is obtained by

$$\begin{array}{c} {\text{w}}_{i}=\sum_{j=1}^{i}(q_{i,j}{\text{w}}_{j}+p_{i}x) \end{array}$$ (1)

where the interlocked learning method recursively updates *q*_*i*,*j*_ and *p*_*i*_. The equation is derived from a Gram-Schmidt orthonormalization procedure [34]. The corresponding eigenvalues λ_*i*_ are calculated by

$$\begin{array}{c} \lambda_{i}^{2}=(\delta \lambda_{i}{)}^{2}+\psi {\text{y}}_{i}(2\delta \lambda_{i}{\text{y}}_{i}+\psi {\text{d}}_{i}{\text{y}}_{i}) \end{array}$$ (2)

with ${\text{y}}_{i}={\text{w}}_{i}^{T}\left(\text{x}-\text{c}\right)$, ${\text{d}}_{i}={\text{d}}_{i-1}-{\text{y}}_{i-1}^{2},{\text{d}}_{1}=||\text{x}-\text{c}|{|}^{2}$. The scalars *δ* and *ψ* = 1 − *δ* are learning rates. The proper choice of the learning rates is crucial since values which are too large cause oscillations, and values which are too small cause the learning algorithm to be caught in local minima. Nevertheless, most learning rates are chosen as simple constants or exponentially decaying terms in neural network-based PCA [17]. In contrast, the learning rate control for *δ* and *ψ* used in this work is based on a variance match between the eigenvalues λ_*i*_ and the neuron output **y**_*i*_. A full derivation is given in S1 Appendix. The change in the center point

$$\begin{array}{c} \text{c}=\text{c}+\delta (\text{x}-\text{c}) \end{array}$$ (3)

also depends on the adaptive learning rate *δ*.

An alternative way to continuously update the set of eigenvectors, eigenvalues and center is the incremental PCA, which is briefly described in the following.

#### Incremental PCA

Incremental PCA algorithms [8, 40] are capable of updating the set of eigenvectors and eigenvalues incrementally. On each data point presentation, the intermediate eigenproblem is solved for that data point. In [8], an incremental PCA approach with adaptive dimensionality adjustment is presented. While a full derivation is out of scope of this work, a brief introduction is necessary to understand the following benchmark. The model is described by an eigenspace model *ω* = {**c**, **W**, **Λ**, *N*}, with **c** being the center point, **W** the matrix containing the eigenvectors in each row, **Λ** the diagonal matrix containing the eigenvalues, and *N* the continuously increasing count of presented data points. The subspace dimensionality is denoted by *m*. Whenever a new data point **x** is presented, the current model is updated without having access to old observations nor their covariance matrix. If necessary, the incremental approach has to be able to increase the dimensionality to *o* = *m* + 1 to achieve a best possible fit. Therefore, the incremental method updates the eigenspace model *ω* = {**c**, **W**, **Λ**, *N* + 1} to an output dimensionality *o*. The eigenspace model is an approximate solution to the eigenproblem

$$\begin{array}{c} \text{CW}=\text{W}\Lambda \end{array}$$ (4)

with **C** being the covariance matrix that is at least conceptually continuously updated by

$$\begin{array}{c} \text{C}=\frac{N}{N+1}\text{C}+\frac{N}{{\left(N+1\right)}^{2}}\xi \xi^{T} \end{array}$$ (5)

with ***ξ*** = **x** − **c**. However, the covariance matrix C is never explicitly computed, only the eigenspace is updated as shown later. The mean is updated continuously as well by

$${\text{c}}^{\prime }=\frac{1}{N+1}(N\text{c}+\text{x})$$ (6)

$$=\frac{N}{N+1}\text{c}+\frac{1}{N+1}\text{x}$$ (7)

where the impact of new data points **x** decays over time to ensure convergence in (5)-(7). The new eigenvectors with an increased dimensionality of *o* = *m* + 1 must be the result of a rotation $\text{R}\in {\mathbb{R}}^{o\times o}$ of the latest eigenvectors **W** complimented by an orthogonal unit vector. This unit vector is chosen to be the residual vector

$$\begin{array}{c} h=\xi -\text{W}{\text{W}}^{T}\xi \end{array}$$ (8)

which is further normalized to $\hat{\text{h}}=\frac{\text{h}}{||\text{h}|{|}_{2}}$ for all **h** ≠ 0 and $\hat{\text{h}}=0$ otherwise. The new eigenvectors

$$\begin{array}{c} \text{W}=[\text{W},\hat{\text{h}}]\text{R} \end{array}$$ (9)

are the rotated old eigenvectors complemented by the residual vector. By substituting (5) and (9) into the eigenproblem (4), the rotation matrix **R** and the eigenvalues **Λ** are obtained

$$\begin{array}{c} (\frac{N}{N+1}\underset{R^{o\times o}}{\underset{︸}{\left[\begin{array}{cc} \Lambda & 0\\ 0^{T} & 0 \end{array}\right]}}+\frac{N}{{\left(N+1\right)}^{2}}\underset{R^{o\times o}}{\underset{︸}{\left[\begin{array}{cc} \text{y}{\text{y}}^{T} & \gamma \text{y}\\ \gamma {\text{y}}^{T} & \gamma^{2} \end{array}\right]}})\text{R}=\text{R}\Lambda \end{array}$$ (10)

with **y** = **W**^*T*^ ***ξ*** and $\gamma ={\hat{\text{h}}}^{T}\xi$ (shown in [8]). The rotation matrix **R** can be used in (9) to obtain the new eigenvectors, while the eigenvalues **Λ** are directly obtained from (10). Based on a given stopping rule, it can be determined if the newly added dimension is necessary or can be discarded.

### Stopping rules in offline PCA

Stopping rules are used for offline PCA to find the optimal number of principal components. The eigenvalues λ_*i*_ are for the following notations stored in a set *V* = {λ_1_, λ_2_, …, λ_*m*_}. One of the most popular methods to determine the optimal number of meaningful dimensions *m* is the eigenvalue-one criterion [44]. The approach follows the idea to keep all eigenvalues λ_*i*_ with a value greater than one. Every eigenvalue λ_*i*_ that fulfills this condition is kept, all eigenvalues λ_*i*_ below that threshold are discarded. The characteristic that makes this method so popular is its simplicity. With the set of eigenvalues *V* = {λ_1_, λ_2_, …, λ_*m*_}, the optimal number of dimensions

$$\begin{array}{c} m=|\{b\in V\;|\;b>1\}| \end{array}$$ (11)

is the number of eigenvalues greater than one. The method results often in retaining the correct number of dimensions when applied to a small data set. In [45], the accuracy of the eigenvalue-one criterion is investigated and it is recommended to apply the method on tasks with 30 or less variables. A problem associated to this method is that the difference between the eigenvalues is not taken into account. For example, if a component has a value of 1.01 and the following has a value of 0.99, the first component is retained while the second is removed. Generally it can be said, that this method is useful for a quick analysis without the requirement of much insight.

Another approach towards finding the optimal number of meaningful components *m* is to retain all eigenvalues greater than the average $\frac{1}{n}\sum_{i=1}^{n}\lambda_{i}$. Each eigenvalue λ_*i*_ that is greater than the average is kept. This method has the same complexity as the eigenvalue-one criterion, just with a different threshold. The output dimension

$$\begin{array}{c} m=|\{b\in V\;|\;b>\frac{1}{n}\sum_{i=1}^{n}\lambda_{i}\}| \end{array}$$ (12)

is defined as the number of eigenvalues λ_*i*_ stored in the set *V* = {λ_1_, λ_2_, …, λ_*m*_} that are larger than the average.

A more complex method to find the optimal number of meaningful principal components is to keep all eigenvalues that are larger than a predefined proportion of the total variance $\lambda_{\text{total}}=\sum_{i=1}^{n}\lambda_{i}$. In the following, *η* ∈ [0, 1] is used to define the proportion of the total variance λ_total_. This factor is used to check if an eigenvalue is larger than $\eta \sum_{i=1}^{n}\lambda_{i}$. This method is more complex than the previous two methods due to the extra parameter *η*. It has to be taken into account that the optimal parameter choice depends on the dimension *n* of the data set and the eigenvalue distribution. For example, when working with a ten-dimensional data set with an exponential eigenvalue decline, the parameter is completely different from a thousand dimensional data set with a linear eigenvalue decline. The optimal output dimensionality

$$\begin{array}{c} m=|\{b\in V\;|\;b>\eta \sum_{i=1}^{n}\lambda_{i}\}| \end{array}$$ (13)

is the number of eigenvalues λ_*i*_ of the set *V* = {λ_1_, λ_2_, …, λ_*m*_} that are greater than the predefined proportion *η* of the total variance λ_total_.

The last reviewed stopping rule is also based on the relative impact of each eigenvalue. Instead of retaining all eigenvalues greater than a certain proportion of the total variance (13), this method is based on the cumulative percentage of the total variance. Therefore, the parameter is introduced *θ* ∈ [0, 1] that is chosen depending on the required fit and the acceptable complexity. A factor towards one would keep almost all components, while a factor close to zero only retains very few components. The optimal number of meaningful components

$$\begin{array}{c} m=\text{arg}\;\underset{z}{\text{min}}\;\{z\in \left\{1,\ldots ,n\right\}\;|\;\sum_{i=1}^{z}\lambda_{i}\geq \theta \sum_{i=1}^{n}\lambda_{i}\} \end{array}$$ (14)

is the smallest *z* that fulfills the above inequality, with the eigenvalues λ_*i*_ and the complexity parameter *θ*. Please note that the eigenvalues λ_*i*_ have to be sorted by size in descending order for this criterion. In general, the computation time of all stopping rules is sped up when the eigenvalues are sorted. Once an eigenvalue is below a stopping-rule-specific threshold, all following eigenvalues are as well below that threshold. In this way the computational overhead is minimized.

### Adaptive online dimensionality adjustment

In traditional offline PCA, the principal components are computed as the eigenvectors of the covariance matrix, which is computationally inefficient for large data sets. Based on the eigenvectors, a stopping rule is applied (11)-(14) to find a lower dimensionality *m* ≤ *n*. Since the entire data set is presented at once, the optimal dimensionality *m* is determined only once. When working with a data stream, the optimal dimensionality may change continuously, requiring a continuous update of *m* to achieve a good fit. While different approaches were presented to continuously adapt the dimensionality *m* [7, 8, 46], they are all limited to an increment of one per presented data point. This prevents the online PCA to take abrupt changes in the data into account. The algorithm proposed in the following is able to adjust the dimensionality by an arbitrary step size in a computationally efficient way; the exact computational complexity is later investigated. To achieve this, it exploits several natural features of neural network-based PCA and properties of the data distribution. The first feature of neural network-based PCA is that the eigenvalues λ_*i*_ are naturally sorted in a descending order. Second, the components are trained in a hierarchical order, ensuring that the most relevant component is trained first. A third characteristic related to real world data is that the variance is not evenly distributed over all principal components. It is more likely that only some features carry variability and a major part is negligible.

The first characteristic is exploited by initializing the PCA output dimensionality with *m* = 2 for the first training cycle. In this way only the two most relevant principal components are trained and the initial matrix size is reduced to *n* × 2 for **W** and 2 × 2 for **Λ**. The two principal components are trained with a neural network-based PCA approach [34]. This PCA method supports the second characteristic by learning the eigenvalues with the highest variance first. In the following, an estimate of the remaining *n* − *m* eigenvalues has to be obtained. Previous work has shown that eigenvalues λ exhibit a behavior which is close to linear in the logarithmic scale on many real-world data sets [47, 48]. This enables the use of a linear regression model to predict the values of the remaining *n* − *m* eigenvalues.

**Fig 2:**
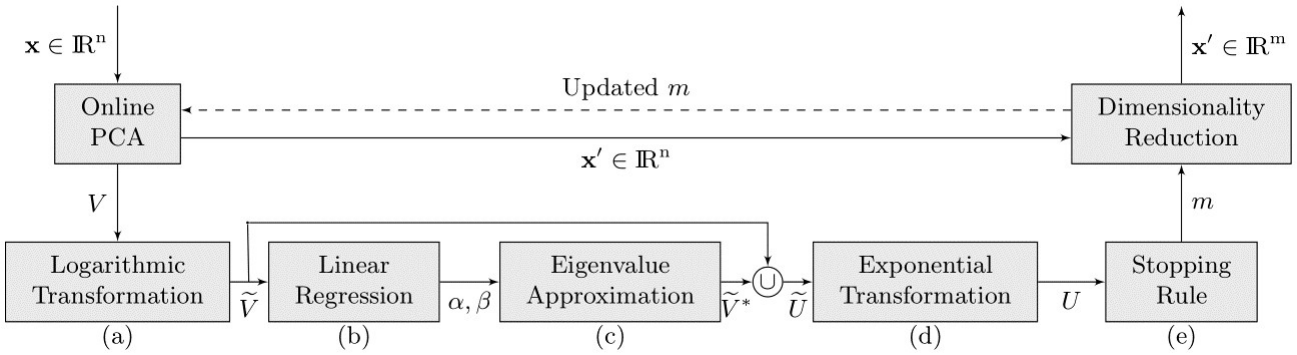
Extended neural network-based PCA workflow with adaptive dimensionality adjustment.

In order to estimate the remaining *n* − *m* eigenvalues, the trained eigenvalues λ_*i*_ (*i* ∈ {1, …, *m*}) contained in the set *V* = {λ_1_, λ_2_, …, λ_*m*_} are first converted into log-eigenvalues

$$\begin{array}{c} {\tilde{\lambda }}_{i}=\text{log}(\lambda_{i}) \end{array}$$ (15)

with $\tilde{V}=\{{\tilde{\lambda }}_{1},{\tilde{\lambda }}_{2},\ldots ,{\tilde{\lambda }}_{m}\}$ being the set of log-eigenvalues (Fig 2a). In the following the tilde denotes logarithmic values. Based on the log-eigenvalues ${\tilde{\lambda }}_{i}$ contained in the set $\tilde{V}$, the slope *α* and the offset *β* of a least-squares regression line in the logarithmic scale are calculated (Fig 2b). To obtain an approximation of the missing *n* − *m* eigenvalues the regression line is extended

$$\begin{array}{c} {\tilde{\lambda }}_{i}^{*}=\alpha i+\beta \end{array}$$ (16)

with *i* ∈ {*m* + 1, …, *n*} and the slope *α* and the offset *β* of the regression line in the logarithmic scale. The star denotes values estimated by the linear regression line. The estimated *n* − *m* log-eigenvalues ${\tilde{V}}^{*}=\{{\tilde{\lambda }}_{m+1}^{*},{\tilde{\lambda }}_{m+2}^{*},\ldots ,{\tilde{\lambda }}_{n}^{*}\}$, generated in step Fig 2c, are supplemented with the original set $\tilde{V}=\{{\tilde{\lambda }}_{1},{\tilde{\lambda }}_{2},\ldots ,{\tilde{\lambda }}_{m}\}$ containing the first *m* trained eigenvalues

$$\begin{array}{c} \tilde{U}=\tilde{V}\cup \left\{{\tilde{\lambda }}_{m+1}^{*},\ldots ,{\tilde{\lambda }}_{n}^{*}\right\}. \end{array}$$ (17)

The elements of the extended set $\tilde{U}=\{{\tilde{\lambda }}_{1},{\tilde{\lambda }}_{2},\ldots ,{\tilde{\lambda }}_{m},{\tilde{\lambda }}_{m+1}^{*},\ldots ,{\tilde{\lambda }}_{n}^{*}\}$ are converted back in step Fig 2d to the non-log domain by applying the exponential function

$$\begin{array}{c} \lambda^{*}=\text{exp}\left({\tilde{\lambda }}^{*}\right) \end{array}$$ (18)

to all real and estimated log-eigenvalues of the set $\tilde{U}$. This yields the set $U=\{\lambda_{1},\lambda_{2},\ldots ,\lambda_{m},\lambda_{m+1}^{*},\ldots ,\lambda_{n}^{*}\}$ in the original space. In the last step (Fig 2e) a chosen stopping rule can be applied to the set *U* to obtain the optimal dimensionality *m*.

**Fig 3:**
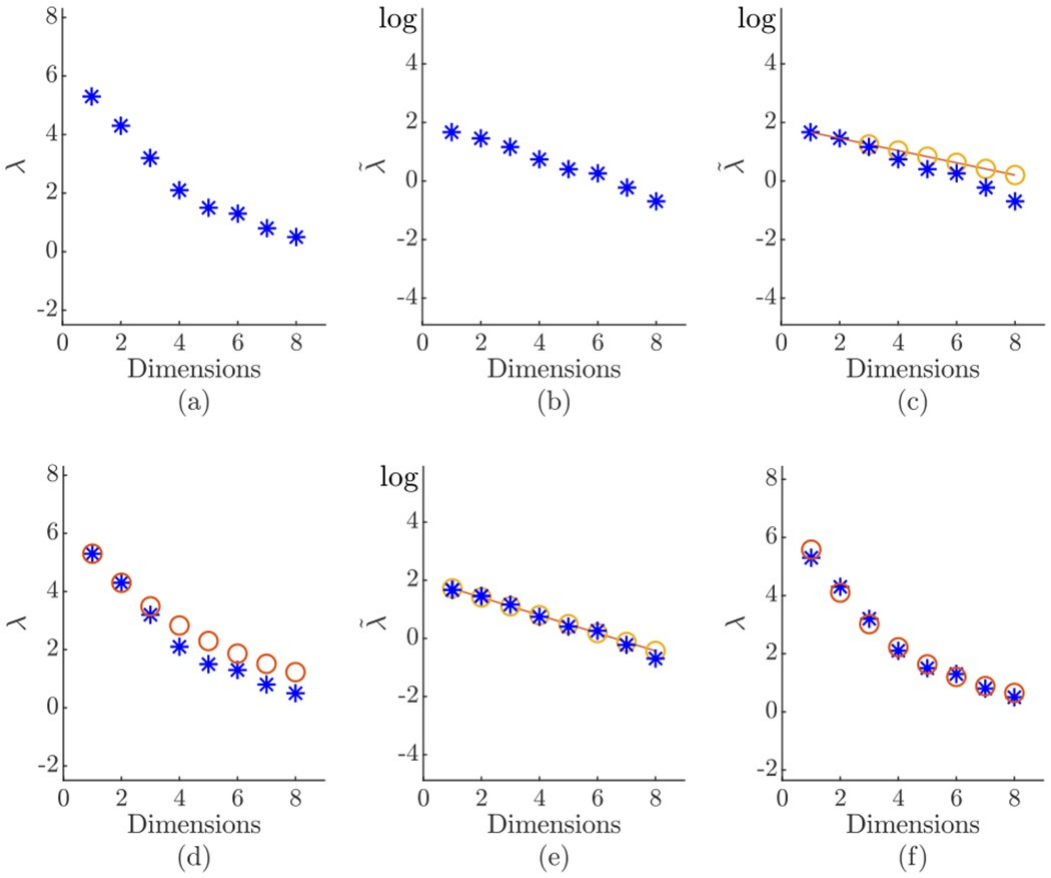
Application of the algorithm to an eight-dimensional Gaussian artificial data set. The real eigenvalues are represented by a star and the estimations by circles: (a-d) 1st step with *m* = 2; (e-f) 2nd step with *m* = 4.

The process for an eight-dimensional synthetic data set is illustrated in Fig 3. The sorted eigenvalues in normal and logarithmic scale are shown in Fig 3a and 3b. In the initial step with *m* = 2, the line of best fit is a simple line through the first two trained logarithmic eigenvalues (Fig 3c) and the estimated log-eigenvalues are transformed back into the normal scale (Fig 3d). Based on these estimations, the dimensionality *m* is adjusted by one of the stopping rules. The newly added eigenvalues (two in this example, thus *m* = 4) are initialized with the estimated values $\lambda_{i}^{*}$ according to the line of best fit and the corresponding eigenvectors with a random orthonormal system. The dimensionality adjustment process is sped up by adding several dimensions at once, giving the method a clear advantage over competing neural network-based and incremental PCA approaches. If the contribution of one or more principal components is not needed to stay above the stopping-rule-specific threshold, the unnecessary dimensions are discarded. The regression parameters are updated based on the extended set after a specific training period (Fig 3e and 3f). With the proposed technique, classical offline stopping rules can be extended for the use in neural network-based PCA.

### Stopping rule online extension

The presented stopping rules (11)-(14) can be rewritten with the proposed method. Therefore, the already trained *m* eigenvalues, with an initial *m* = 2, and the regression parameters *α* and *β* obtained from the *m* trained log-eigenvalues are needed. The total variance is approximated using

$$\begin{array}{c} \lambda_{\text{total}}=\sum_{i=1}^{n}\lambda_{i}\approx \sum_{i=1}^{m}\lambda_{i}+\sigma^{2} \end{array}$$ (19)

by the already trained *m* eigenvalues λ_*i*_ and the residual variance *σ*^2^ [39], both getting updated in every PCA update step.

In case of the eigenvalue-one criterion, (11) can be rewritten by

$$\begin{array}{c} m=|\{b\in U\;|\;b>1\}| \end{array}$$ (20)

as an equation based on the extended set *U* instead of the fully trained set *V*. All real eigenvalues λ_*i*_ and the eigenvalue approximations $\lambda_{i}^{*}$ that are larger than one are kept and will be trained further.

The same process is applied to the approach of keeping all eigenvalues larger than the average (12). The output dimensionality

$$m=|\{b\in U\;|\;b>\frac{1}{n}\lambda_{\text{total}}\}|$$ (21)

depends on the extended set *U* and the adapting average (19). Instead of the static threshold used in the offline version (12), the average $\frac{1}{n}\lambda_{\text{total}}$ is updated with every PCA step. Due to the moving threshold, this method is more complex than the extended eigenvalue-one criterion (20).

For the approach of keeping all eigenvalues greater than a proportion *η* of the total variance (19), the output dimensionality becomes

$$\begin{array}{c} m=|\{b\in U\;|\;b>\eta \lambda_{\text{total}}\}|. \end{array}$$ (22)

The factor *η* offers the possibility to move the threshold at will. The complexity increases due to the moving total variance and the parameter *η*.

In the last case, the dimensionality

$$m=\text{arg}\;\underset{z}{\text{min}}\;\{z\in \left\{1,\ldots ,n\right\}\;|\;\sum_{i=1}^{z}U_{i}\geq \theta \lambda_{\text{total}}\}$$ (23)

can be rewritten as an inequality depending on the already trained *m* eigenvalues λ_*i*_ and the associated reconstructions expressed by the line of best fit parameters *α* and *β*. The method (23) is able to represent all possibly occurring principal component distributions in a robust manner, as long as the eigenvalues are sorted in descending order (which lies in the nature of hierarchical online PCA algorithms).

One extreme would be a fully symmetric data distribution with all dimensions carrying the same variance. Based on the first two principal components, a line of best fit with a slope *α* of zero would perfectly estimate the remaining principal components. On the other hand, a distribution with all variance located in one dimension is also easy to estimate. A large negative slope *α* in combination with the behavior of the exponential function would estimate a value close to zero for all other principal components. In this way, a simple line in a logarithmic scale can approximate many possibly occurring eigenvalue distributions in the normal scale.

### Influence of the eigenvalue distribution

With the presented approach, stopping rules were extended towards neural network-based PCA. Due to the initialization of the eigenvalues λ_1,2_ with a random value and the weights of the eigenvectors with a random orthonormal system, the stopping rules cannot be applied right away. The immediate application of the dimensionality adjustment to the random eigenvalues would yield a random output dimension *m*. Therefore, the neural network-based PCA has *Γ* update steps in the beginning to train the two initial eigenvalues λ_1,2_, before the dimensionality adjustment is activated. This hyperparameter is equal to a pretraining on a small batch data set and does not require special tuning.

**Fig 4:**
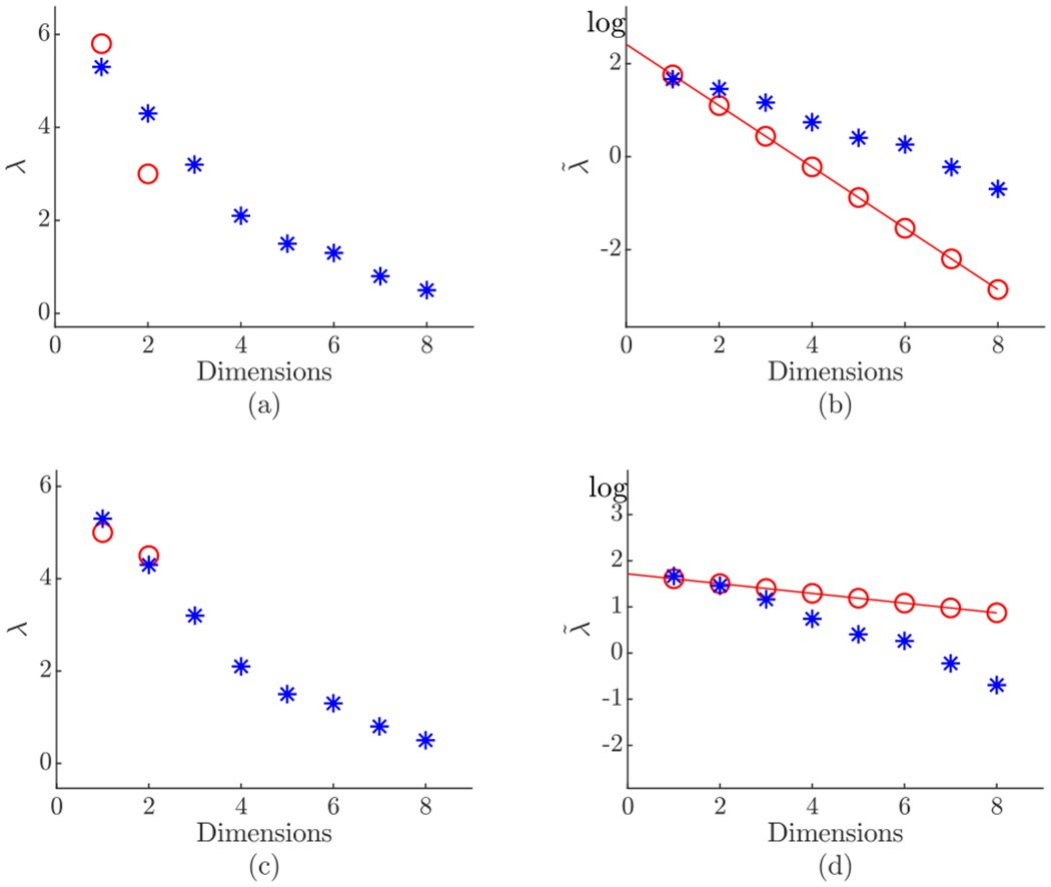
Occurrence of over- or underestimations due to not fully trained eigenvalues λ_1,2_. The real eigenvalues are represented by a star (*) and the estimations by circles (o): (a)-(b) shows an underestimation which results in a sharply declining line of best fit; (c)-(d) shows an overestimation which results in an almost flat line of best fit.

To show the possible error potential in the beginning and the necessity of *Γ* an example is given in Fig 3. It is assumed that the two initial eigenvalues λ_1,2_ are not fully trained when the dimensionality adjustment is activated. In the first scenario in Fig 4a, the two initial eigenvalues, represented by circles, are not trained correctly when the dimensionality adjustment is activated. This results into a sharply declining line of best fit in the logarithmic scale (Fig 4b). The algorithm now assumes that all approximations contribute less variance than they actually do. Depending on the stopping rule applied this has different effects. In case of the eigenvalue-one criterion (20) fewer eigenvalues would be above the threshold of one. This leads to a slow adjustment process. The same holds for the percentage of total variance criterion (22) because fewer eigenvalues would be above the specific threshold *η*. On the other hand, a sharp declining line would result in a drastically increase in dimensionality for the eigenvalue-average criterion (21). With less variance attributed, more dimensions are required to reach a certain threshold of the total variance λ_total_. The same effect is seen for the cumulative percentage of total variance criterion (23) since more eigenvalues are needed to represent a certain amount of the total variance.

In Fig 4c another initial distribution is shown which leads to an almost flat line of best fit (Fig 4d). In this case too much variance is attributed to the eigenvalues. This leads to a behavior that is exactly the opposite compared to the sharply declining line.

This demonstrates that the dimensionality adjustment process is sensitive to a premature start, which will be in depth investigated in the comprehensive study carried out in this work. The impact of the two initial eigenvalues λ_1,2_ presented in Fig 4 shows the need of Γ PCA update steps before the activation of the dimensionality adjustment.

The quality of the linear regression model in the logarithmic scale is limited by the underlying data distribution. The best fit is achieved when the eigenvalues have an exponential decline. This leads to a straight line in the logarithmic scale. Nevertheless, there is no perfectly exponentially declining eigenvalue distribution and therefore a small error between real eigenvalues and estimations is expected.

**Algorithm 1** Online PCA dimensionality adjustment procedure based on (15)-(23)

**Input**: current dimensionality *m*, current eigenvectors **W**, current eigenvalues **Λ**, current center **c**, new input **x**

**Output**: updated dimensionality *m*, updated eigenvectors **W**, updated eigenvalues **Λ**, updated center **c**

1: **c**, **W**, **Λ** ← Online PCA(**c**, **W**, **Λ**, **x**, *m*)             ⊳ [34]

2: **if** *κ* > Γ **then**

3:  **procedure** *m* ← DIMENSIONALITY ADJUSTMENT(Λ)

4:   $\tilde{V}\leftarrow \text{Log}\;\text{Transformation}\;({\text{diag}}^{-1}(\Lambda ))$      ⊳ (15)

5:   $\alpha ,\beta \leftarrow \text{Linear}\;\text{Regression}\;(\tilde{V})$           ⊳ (16)

6:   $\tilde{U}\leftarrow \text{Log}\;\text{Eigenvalue}\;\text{Estimation}\;(\alpha ,\beta )$       ⊳ (17)

7:   *U* ← Normal Transformation ($\tilde{U}$)            ⊳ (18)

8:   *m* ← Stopping Rule (*U*)                 ⊳ (20)-(23)

9:  **end procedure**

10: **end if**

11: *κ* = *κ* + 1

### Algorithm overview

To provide an overview of the proposed method for adaptive dimensionality adjustment in neural network-based PCA: The algorithm 1 has as input parameters the current dimensionality *m* (which is initially set to 2), the current eigenvectors **W**, the current eigenvalues **Λ**, the current center **c** and the new input data **x**. Outputs are the updated dimensionality *m*, the updated eigenvectors **W**, the updated eigenvalues **Λ** and the updated center **c**. The function is called when-ever a new data point **x** is presented. With this data point a full training cycle *κ* ∈ *K* is carried out by first updating the PCA parameters and then applying the dimensionality adjustment approach. In the first step, the hierarchical PCA model parameters are updated [34] and then the presented approach for adaptive dimensionality adjustment is applied. However, in the beginning the initial dimensionality is set to two, and the first two principal components λ_1,2_ are trained for Γ training cycles, before dimensionality adjustment is activated for the first time. This hyperparameter is introduced to make the initial training phase more stable and is comparable with a pretraining with a batch PCA on a small data set. Therefore, this parameter does not require any special tuning. Once this initial learning phase is over, the dimensionality adjustment procedure is started. Based on the set of log-eigenvalues $\tilde{V}$ (15), the linear regression parameters *α*, *β* are updated (16), (17). The parameters are used in the following to approximate the remaining *n* − *m* eigenvalues in the logarithmic scale and merge them with the already existing *m* log-eigenvalues (18). The presented stopping rules are applied on the non-log set *U* (20)-(23), providing an updated dimensionality *m*.

In the following, neural network-based PCA with the extended stopping rules and the adaptive dimensionality adjustment are applied to a variety of data sets. In order to rate the quality of each stopping rule, the data sets were chosen to be different from each other in as many aspects as possible. The obtained results are presented in a comprehensive study. The algorithms performance is additionally benchmarked against a competitive incremental PCA approach.

## Results and discussion

In the following it is tested if the presented approach can adaptively estimate the correct final dimensionality for different data sets. All extended stopping rules (20)-(23) are considered in this study. A goal of this comprehensive experimental study is to demonstrate the quality of the online PCA with the extended stopping rules on a variety of data sets. The data sets (Table 1) differ from each other e.g. in dimensionality and eigenvalue distribution, covering a broad spectrum of data characteristics occurring in real world applications.

**Table 1:** Data set properties and training parameters.

Data set		Dimensionality (*n*)	Instances (*K*)	Parameter Γ	Type
Cameraman	[49]	64	1024	100	Image
Circles	[49]	64	1024	100	Image
PHM08	[50]	26	321	10	Sensor
CareerCon19	[51]	10	1280	100	Sensor
Waveform	[52]	40	5000	100	Synthetic
Waveform2	[52]	21	5000	100	Synthetic

The first two data sets are grayscale images of a cameraman [49] and circles [49]. The images with a size of 256 × 256 pixel are decomposed into 1024 non-overlapping blocks with a size of 8 × 8. The 64-dimensional images are used to test the proposed algorithm (*n*_1,2_ = 64). The PHM08 data set [50] is a real world data set containing several sensor channels (*n*_3_ = 26) describing the degradation of a turbofan engine. The CareerCon19 data set [51] contains orientation, velocity and acceleration data (*n*_4_ = 10) of a real robot driving over a surface. The fifth data set contains synthetic waveform data [52]. The data set is augmented by 19 Gaussian noise dimensions. It is distinguished between the full data set (*n*_5_ = 40) called waveform and a modified version waveform2 without the noise dimensions (*n*_6_ = 21).

The data sets are treated as if the data points **x** occur online in a random order and a data point only occurs once. The number of total training cycles *K* is the number of instances a data set has. A single training cycle *κ* ∈ {1, …, *K*} consists of the online PCA update step and the dimensionality adjustment. All online stopping rules (20)-(23) are tested on each of the presented data sets (Table 1). The dimensionality adjustment process is activated Γ training cycles after the start, which means that the online PCA has Γ training cycles to train the randomly initialized eigenvalues λ_1,2_. Due to the small number of instances contained in the PHM08 data set, the parameter Γ is smaller compared to the Γ used for all other data sets. Using a Γ = 100 for the PHM08 data set would falsify the results because the dimensionality adjustment process would start after a third of the data points has already been presented. The parameters keep their values for all stopping rules and only vary over the data sets. Every stopping rule and data set combination is tested and repeated 100 times with the same parameter set.

The extended stopping rules using a linear regression model in logarithmic scale are compared to a standard offline PCA based on singular value decomposition together with the classical offline stopping rules. The eigenvalues and weights are updated with an hierarchical online PCA [34] algorithm. The aim is to estimate the correct final dimensionality long before all data points are represented. Hence, it is tested if the different online stopping rule approaches are able to determine the correct dimensionality *m* before all data points (25%, 50%, 75%, 100%) of a data set are presented. The stopping rules are tested in the order of increasing complexity. The results are presented with the mean *μ* and the corresponding standard deviation *s* calculated over 100 repetitions. It is assumed that the calculated final dimensionality is correct if the standard deviation *s* is adequately small and within the difference of the mean *μ* and the offline PCA reference. The results are additionally benchmarked against competing algorithms.

### Eigenvalue-one criterion

The first approach tested on the data sets (Table 1) is the online version of the eigenvalue-one criterion (20). The results of the online dimensionality adjustment process with the online eigenvalue-one criterion (20) are compared to the classical offline PCA with the eigenvalue-one criterion (11). Additionally, it is observed how many data points **x** have to be presented to predict the correct final dimensionality *m*. The eigenvalue-one criterion is straightforward in contrast to the other methods by comparing the existing eigenvalues λ_*i*_ and the approximations $\lambda_{i}^{*}$ with the fixed threshold of one. Every eigenvalue larger than the threshold of 1 is further trained. The results are presented in Table 2.

**Table 2:** Results obtained with the online eigenvalue-one criterion compared with the offline version.

	Data set	*μ* ± *s*
a)	Cameraman_25%_	1.0±0.0
	Cameraman_50%_	1.0±0.0
	Cameraman_75%_	1.0±0.0
	Cameraman_100%_	1.0±0.0
	Offline PCA	1
b)	Circles_25%_	1.0±0.0
	Circles_50%_	1.0±0.0
	Circles_75%_	1.0±0.0
	Circles_100%_	1.0±0.0
	Offline PCA	1
c)	PHM08_25%_	13.9±8.2
	PHM08_50%_	13.1±8.7
	PHM08_75%_	12.0±8.7
	PHM08_100%_	11.2±8.7
	Offline PCA	8
d)	CareerCon19_25%_	3.3±1.7
	CareerCon19_50%_	2.8±0.9
	CareerCon19_75%_	2.8±0.8
	CareerCon19_100%_	2.9±0.8
	Offline PCA	3
e)	Waveform_25%_	8.6±2.2
	Waveform_50%_	7.4±2.0
	Waveform_75%_	6.5±1.8
	Waveform_100%_	6.2±1.6
	Offline PCA	20
f)	Waveform2_25%_	3.6±0.8
	Waveform2_50%_	3.5±0.7
	Waveform2_75%_	3.3±0.6
	Waveform2_100%_	3.2±0.4
	Offline PCA	12

The two image data sets (Table 2a and 2b) both only have one eigenvalue larger than the threshold. In both cases, the correct final dimensionality with a standard deviation of *s* = 0 is achieved after 25% of the data points are presented. It has to be noted, that the calculation of the line of best fit needs at least two eigenvalues. This means, that the algorithm recommends to use only one, but actually keeps the second eigenvalue for further approximations.

**Fig 5:**
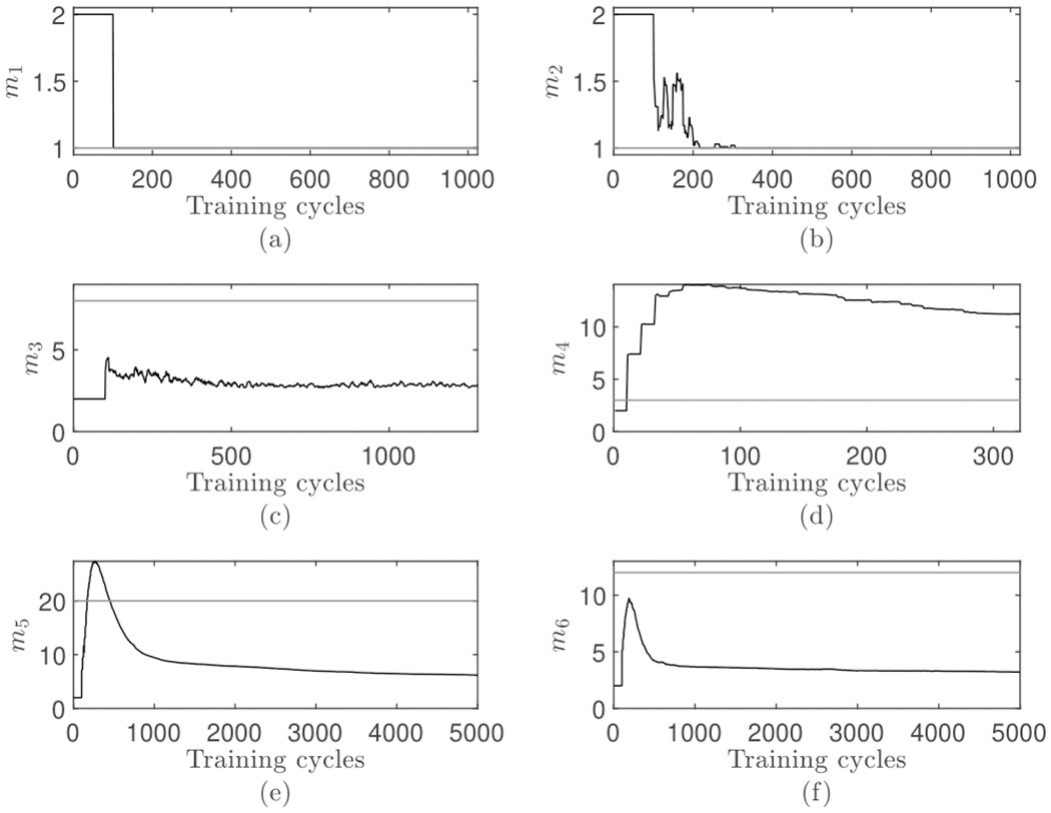
Visualization of the dimensionality adjustment process for the online eigenvalue-one criterion: (a-f) Adjustment process corresponds to the results in Table 2. Each data point in each time series is the mean value of all 100 repetitions. The horizontal reference line represents the optimal dimensionality.

For the sensor data sets (Table 2c and 2d), the online eigenvalue-one criterion estimates a correct mean with a sufficiently small standard deviation for the CareerCon19 data set. For the higher-dimensional PHM08 data set the correct final dimensionality was not achieved due to the characteristic of the approach of only training eigenvalues larger than the threshold. With certain underlying data distributions the regression model may overestimate the eigenvalues (Fig 4) and therefore a stopping rule takes less components than actually needed. The same effect is seen for the synthetic waveform data sets (Fig 5e and 5f). In both cases the approximated dimensionality is below the real dimensionality which indicates that the linear regression model is resulting in a sharply declining line approximating a value below the threshold of one. Additionally, it is assumable that the logarithmic eigenvalue distribution is not linear.

The adjustment process for all data sets is shown in Fig 5. Early on, when the first eigenvalues λ_1,2_ are not fully trained, the dimensionality seems to overshoot on some data sets (e.g. Fig 5c). Once the online PCA has seen enough points to correctly update the eigenvalues, the dimensionality approximation adapts quickly to its final value.

In summary, the overall quality of the online dimensionality adjustment in combination with the eigenvalue-one stopping rule seems to get worse, the higher the dimensionality of the data set is. This is related to the underlying data distribution and the fixed threshold.

### Eigenvalue-average criterion

The second approach tested is the extended eigenvalue-average criterion (21). In comparison to the offline eigenvalue-average criterion (12), the online version of the eigenvalue-average criterion has a moving threshold that is updated in every training cycle *κ*. In this way, the online eigenvalue-average criterion also differs from the previously analyzed eigenvalue-one criterion with a fixed threshold. The results are presented in Table 3, comparing the online criterion combined with the online PCA against the offline versions.

**Table 3:** Comparison of the online eigenvalue-average criterion with the offline version.

	Data set	*μ* ± *s*
a)	Cameraman_25%_	1.8±1.0
	Cameraman_50%_	1.7±0.5
	Cameraman_75%_	1.6±0.5
	Cameraman_100%_	1.6±0.4
	Offline PCA	1
b)	Circles_25%_	1.7±1.0
	Circles_50%_	1.3±0.5
	Circles_75%_	1.2±0.5
	Circles_100%_	1.1±0.4
	Offline PCA	1
c)	PHM08_25%_	2.2±0.4
	PHM08_50%_	2.0±0.1
	PHM08_75%_	2.0±0.0
	PHM08_100%_	2.0±0.0
	Offline PCA	3
d)	CareerCon19_25%_	2.8±0.7
	CareerCon19_50%_	2.5±0.6
	CareerCon19_75%_	2.6±0.5
	CareerCon19_100%_	2.6±0.6
	Offline PCA	3
e)	Waveform_25%_	5.2±0.6
	Waveform_50%_	5.2±0.6
	Waveform_75%_	5.2±0.6
	Waveform_100%_	5.2±0.5
	Offline PCA	2
f)	Waveform2_25%_	2.4±0.7
	Waveform2_50%_	2.4±0.6
	Waveform2_75%_	2.3±0.5
	Waveform2_100%_	2.4±0.5
	Offline PCA	2

The results show that the criterion estimates a mean *μ* close to the correct final dimensionality, with a sufficiently small standard deviation *s* for all but the waveform data set. The waveform data set is augmented by 19 Gaussian noise dimensions reducing the efficiency of the linear regression model in log scale. This affects the average and thus the threshold. Due to the error between true and approximated eigenvalues and the underlying data distribution, the correct dimensionality could not be obtained for the waveform data set. The eigenvalue-average criterion was able to calculate the final dimensionality before all data points are presented. Already after 25% the estimated dimensionality was close to the real dimensionality (shown by the small remaining standard deviation *s*) proving the fast adaptation process and robustness, as long the noise dimensions contribute a small portion of the total variance.

**Fig 6:**
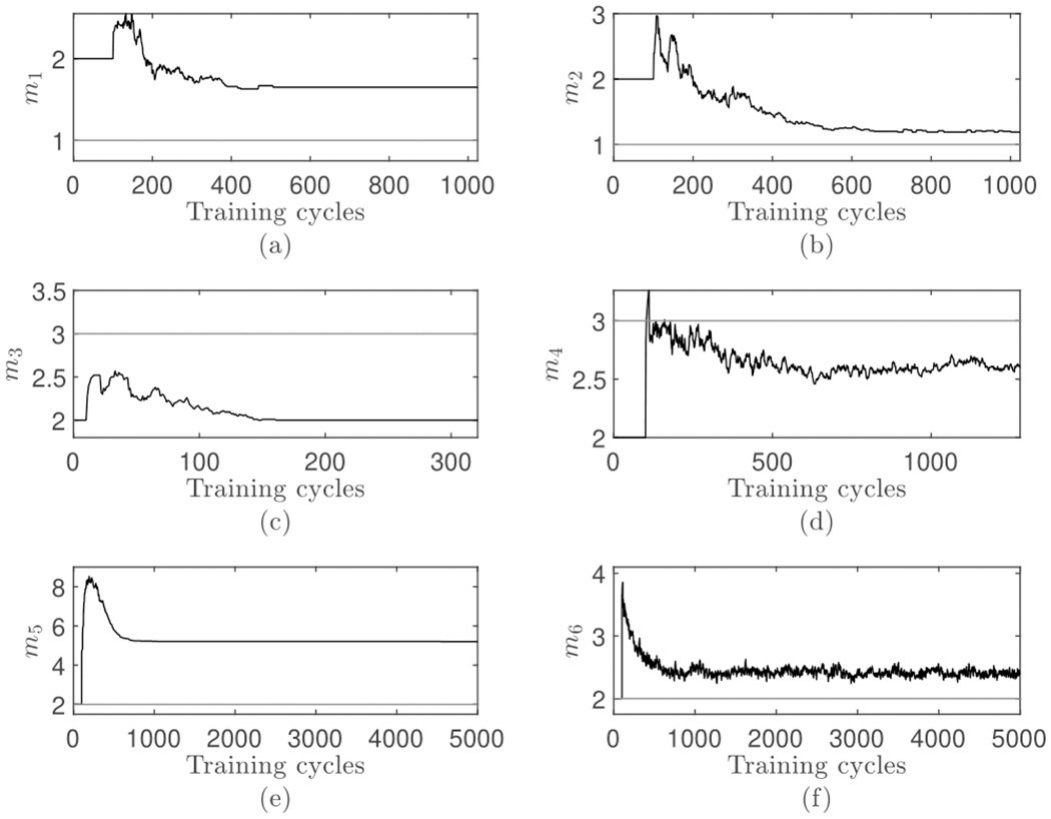
Visualization of the dimensionality adjustment process for the online eigenvalue-average criterion applied on the presented data sets (Table 1): (a-f) Adjustment process corresponds to the results in Table 3. Each data point in each time series is the mean value of all 100 repetitions. The horizontal reference line represents the optimal dimensionality.

The adjustment process is shown in Fig 6. A small overshot occurs after activating the dimensionality adjustment for all data sets but is corrected quickly. In all cases, the process is quickly moving towards a final dimensionality which is then held with only small or no variation.

In comparison to the eigenvalue-one criterion analyzed previously, the moving threshold improves the quality drastically. While the online eigenvalue-one criterion had problems to approximate the correct dimensionality due to its simplicity, the results obtained with the online eigenvalue-average criterion shows a mean close to the real dimensionality with a sufficiently small standard deviation for all but the waveform data set.

### Percentage of total variance criterion

The next stopping rule that is extended towards online PCA keeps all eigenvalues that contribute more than a certain percentage of the total variance (22). In comparison to the approaches considered before, this stopping rule increases the complexity further by adding an extra factor *η* to the moving threshold. The results are shown in Table 4, only keeping the eigenvalues larger than *η* = {0.01, 0.025, 0.05} of the total variance.

**Table 4:** Percentage of total variance criterion with different threshold accuracies: *η*_1_ = 0.01, *η*_2_ = 0.025, *η*_3_ = 0.05.

	Data set	*μ* ± *s*		
		*η*_1_	*η*_2_	*η*_3_
a)	Cameraman_25%_	2.6±1.3	1.3±0.6	1.0±0.2
	Cameraman_50%_	2.0±0.7	1.2±0.4	1.0±0.0
	Cameraman_75%_	2.0±0.6	1.2±0.4	1.0±0.0
	Cameraman_100%_	2.0±0.6	1.2±0.4	1.0±0.0
	Offline PCA	5	3	2
b)	Circles_25%_	2.3±1.8	1.4±0.8	1.2±0.7
	Circles_50%_	1.6±1.0	1.0±0.2	1.0±0.1
	Circles_75%_	1.5±0.6	1.0±0.2	1.0±0.0
	Circles_100%_	1.4±0.5	1.0±0.2	1.0±0.0
	Offline PCA	3	3	1
c)	PHM08_25%_	2.7±1.0	2.4±1.1	2.2±0.5
	PHM08_50%_	2.6±0.5	2.1±0.3	2.0±0.1
	PHM08_75%_	2.6±0.5	2.0±0.2	2.0±0.0
	PHM08_100%_	2.6±0.5	2.0±0.1	2.0±0.0
	Offline PCA	2	2	2
d)	CareerCon19_25%_	6.2±1.2	5.0±0.8	3.9±1.3
	CareerCon19_50%_	5.9±0.8	4.8±0.8	3.8±1.1
	CareerCon19_75%_	5.9±0.9	4.8±0.7	3.9±1.2
	CareerCon19_100%_	5.8±0.8	4.7±0.5	3.8±1.1
	Offline PCA	5	5	3
e)	Waveform_25%_	22.2±1.9	2.4±0.7	2.0±0.0
	Waveform_50%_	22.2±1.9	2.4±0.7	2.0±0.0
	Waveform_75%_	22.2±1.9	2.5±0.7	2.0±0.0
	Waveform_100%_	22.2±1.9	2.3±0.7	2.0±0.0
	Offline PCA	40	2	2
f)	Waveform2_25%_	20.5±1.0	2.8±0.6	2.2±1.1
	Waveform2_50%_	20.7±0.8	2.9±0.5	2.0±0.2
	Waveform2_75%_	20.8±0.6	2.9±0.7	2.0±0.2
	Waveform2_100%_	20.9±0.5	2.9±0.5	2.0±0.2
	Offline PCA	20	2	2

For the image data sets, the approximated dimensionality converged to a mean *μ* that is smaller than correct dimensionality. The PHM08, CareerCon19 and waveform2 results are within the correct range and have a small remaining standard deviation. The only wrong dimensionality prediction is found at the waveform data set with a factor of *η*_1_ = 0.01. The 19 noise dimensions together contribute more than 1% of the total variance. Hence, the eigenvalue distribution changes abruptly and the linear regression model yields a big error between true values and approximations. If the approximations underestimate the true values, it may occur that they fall below that 1% threshold and are not trained further. Once the contribution of each noise dimension falls below the threshold *η*, a correct dimensionality approximation is achieved.

For most data set and *η* combinations, the correct final dimensionality is achieved way before all data points were presented, demonstrating that this online stopping rule in combination with the linear regression model in logarithmic scale is well applicable.

**Fig 7:**
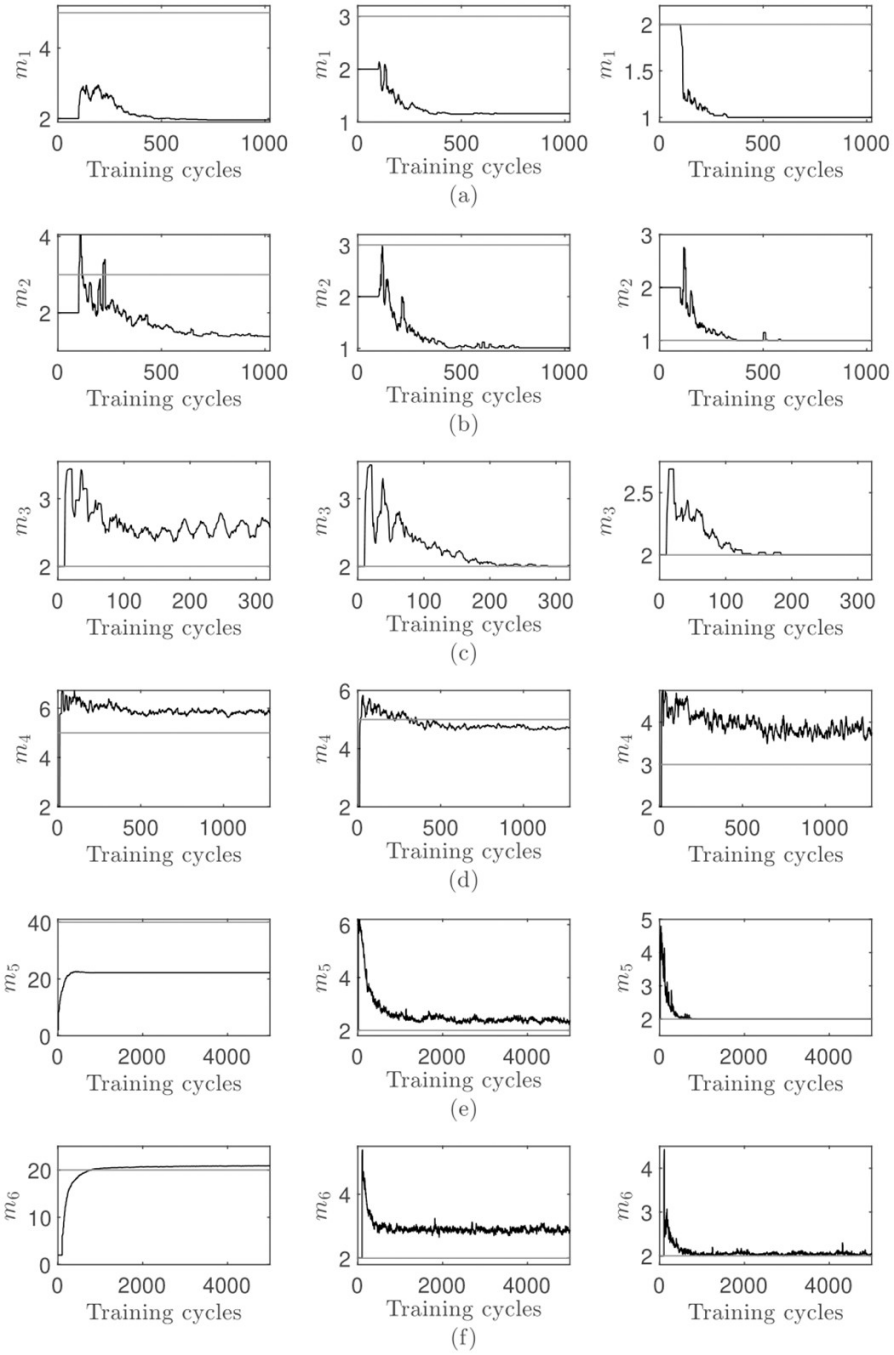
Visualization of the results achieved with the online percentage of the total variance criterion. The first plot in each row has an accuracy factor of *η*_1_ = 0.01, the middle plot *η*_2_ = 0.025 and the right plot *η*_3_ = 0.05: (a-f) shows the results obtained on each data set, with the data set order corresponding to Table 4. Each data point in each time series is the mean value of all 100 repetitions. The horizontal reference line represents the optimal dimensionality.

The dimensionality adjustment process is shown in Fig 7. In nearly all data set and parameter combinations, the method is immediately heading towards the final dimensionality. In some cases a small overshot is noticeable, whereas in other cases the dimensionality is slowly adapting without an overshot. This can be explained by the impact of the two initial eigenvalues in the first training cycle *κ* after activating the dimensionality adjustment process (Fig 4). It has to be noted that the first plot in row Fig 7e is showing convergence towards the wrong final dimensionality due to the noise dimensions.

In comparison to the two online stopping rules previously analyzed, this method has a smoother adjustment process. In more cases is the correct final dimensionality achieved. Additionally, the final value is in a smaller range which is proven by the small standard deviation *s*.

### Cumulative percentage of total variance criterion

The last investigated stopping rule is the percentage of total variance criterion (23). It has the same structure as the previous stopping rule (22).

The approach is applied to the given data sets (Table 1) with different proportions *θ* = {0.7, 0.8, 0.9, 0.99}. The results are presented in Table 5. For all four predetermined proportions the approach gets close to the correct final dimensionality for the image data sets. Nevertheless, the standard deviation *s* for the circle data set with *θ* = 0.99 is comparatively large. This behavior occurs when a large number of eigenvalues represent the same variance. The approximation is less stable in this situation. The results for the PHM08 data set are flawless with a correct final dimensionality in all repetitions and a remaining standard deviation *s* close to zero. For the cases *θ* = [0.7, 0.8] the suggested dimensionality is one, but a second component is kept for the regression model. For the other three data sets the calculated dimensionality gets very close to the correct value of one. It is noticeable that the dimensionality has almost fully converged after presenting 25% of the data. Additionally, the standard deviation *s* is small compared to the other stopping rules.

**Table 5:** Cumulative percentage of total variance: *θ*_1_ = 0.7, *θ*_2_ = 0.8, *θ*_3_ = 0.9, *θ*_4_ = 0.99.

	Data set	*μ* ± *s*			
		*θ*_1_	*θ*_2_	*θ*_3_	*θ*_4_
a)	Cameraman_25%_	1.0±0.2	1.7±0.9	3.4±1.6	16.0±1.8
	Cameraman_50%_	1.0±0,0	1.4±0.5	2.9±1.7	26.2±1.2
	Cameraman_75%_	1.0±0.0	1.3±0.5	2.6±1.1	24.3±1.6
	Cameraman_100%_	1.0±0.0	1.3±0.4	2.6±1.0	29.4±1.4
	Offline PCA	1	1	4	30
b)	Circle_25%_	1.1±0.3	1.4±0.7	2.2±1.4	9.4±3.0
	Circle_50%_	1.0±0.0	1.1±0.3	1.8±1.0	17.6±6.5
	Circle_75%_	1.0±0.0	1.0±0.1	1.7±0.5	20.3±11.1
	Circle_100%_	1.0±0.0	1.0±0.0	1.9±0.5	22.0±12.5
	Offline PCA	1	1	2	23
c)	PHM08_25%_	1.0±0.0	1.0±0.0	2.0±0.1	2.6±0.7
	PHM08_50%_	1.0±0.0	1.1±0.0	2.0±0.0	2.0±0.2
	PHM08_75%_	1.0±0.0	1.0±0.0	2.0±0.0	2.0±0.0
	PHM08_100%_	1.0±0.0	1.0±0.0	2.0±0.0	2.0±0.0
	Offline PCA	1	1	2	2
d)	CareerCon19_25%_	2.3±0.5	3.1±0.8	3.4±0.7	5.0±0.0
	CareerCon19_50%_	2.2±0.4	2.8±0.5	3.1±0.4	5.0±0.0
	CareerCon19_75%_	2.0±0.3	2.9±0.5	3.0±0.2	5.0±0.0
	CareerCon19_100%_	2.0±0.2	2.9±0.5	3.0±0.2	5.0±0.0
	Offline PCA	2	3	3	5
e)	Waveform_25%_	18.4±2.5	26.1±2.1	33.6±3.6	39.7±0.8
	Waveform_50%_	19.1±2.0	26.4±1.9	33.6±1.8	39.8±0.8
	Waveform_75%_	19.1±2.0	26.4±1.9	33.6±1.8	39.8±0.7
	Waveform_100%_	19.0±2.1	26.3±1.9	33.6±1.8	39.8±0.7
	Offline PCA	18	25	33	40
f)	Waveform2_25%_	6.0±1.6	11.0±0.6	16.1±0.3	21.0±0.1
	Waveform2_50%_	5.9±1.5	11.0±0.2	16.1±0.3	21.0±0.1
	Waveform2_75%_	6.0±1.4	11.0±0.2	16.0±0.2	21.0±0.0
	Waveform2_100%_	5.8±1.1	11.0±0.1	16.0±0.1	21.0±0.0
	Offline PCA	6	11	16	21

**Fig 8:**
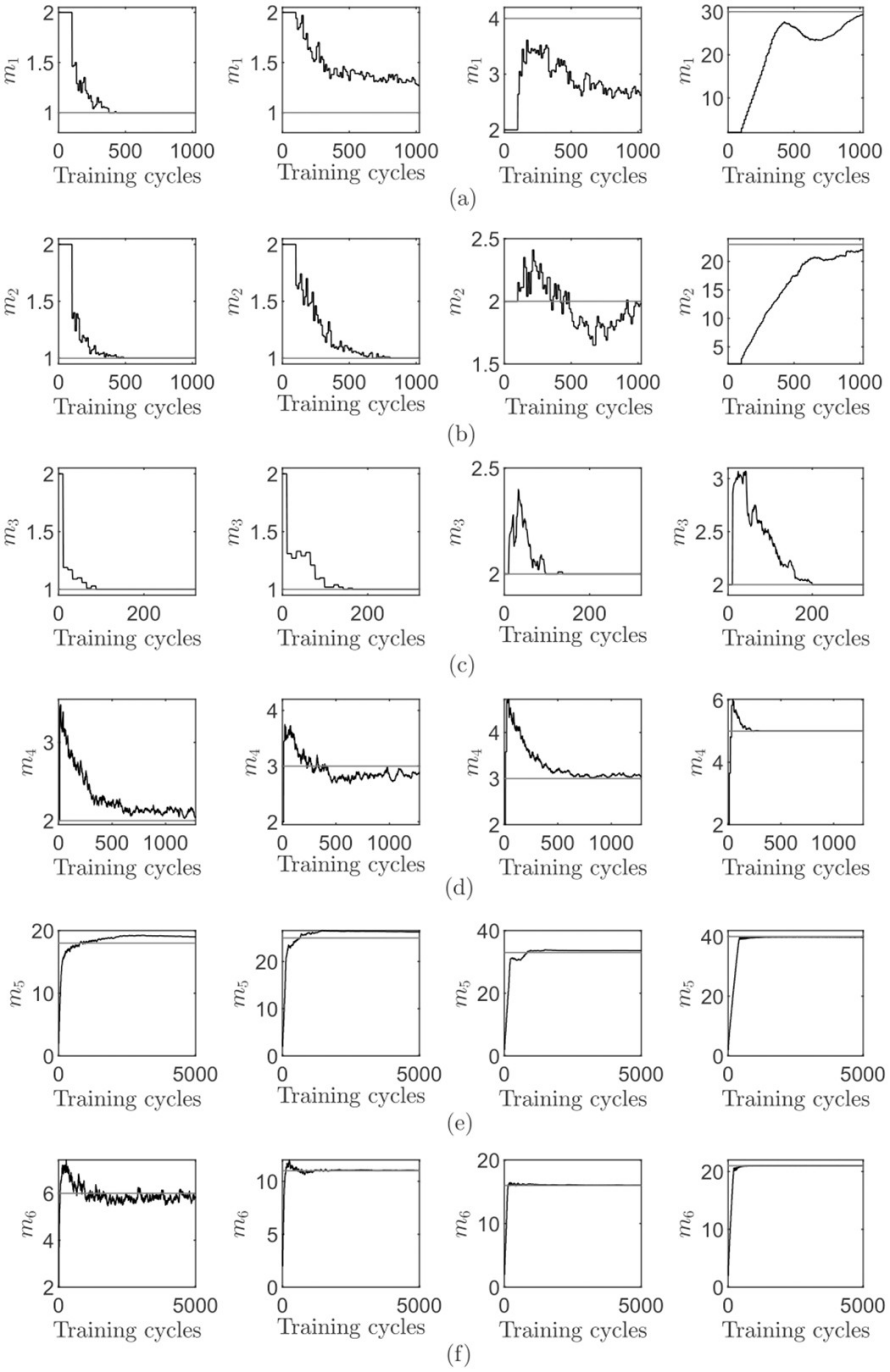
Visualization of the results achieved with the online cumulative percentage of total variance criterion. The first plot in each row has a proportion of *θ*_1_ = 0.70, the left-middle plot of *θ*_2_ = 0.80, the right-middle plot of *θ*_3_ = 0.90 and the right plot of *θ*_4_ = 0.99: (a-f) shows the results obtained on each data set, with a data set order corresponding to Table 5. Each data point in each time series is the mean value of all 100 repetitions. The horizontal reference line represents the optimal dimensionality.

Fig 8 shows the dimensionality adjustment process for the different data sets and proportion combinations. Overall, the adjustment process is smooth. Depending on the underlying data distribution, a small overshot occurs sometimes, e.g. for the CareerCon19 (Fig 8d) data set. After the initial overshot, the final dimensionality is quickly reached. For other data sets, e.g. the waveform data set in Fig 8e, a smaller but steady rise towards the final dimensionality is shown. It is noticeable that the dimensionality is often increased in larger steps, speeding up the adjustment process.

In comparison to the towards online PCA extended stopping rules that were reviewed previously, the cumulative percentage of total variance criterion yields the best results. It is the only approach that successfully determines the correct final dimensionality regardless of the underlying data distribution, the dimensionality and noisy dimensions.

### Benchmark comparison with competing algorithms

The novel approach presented in this work uses a linear regression model in the logarithmic scale to continuously estimate the number of meaningful principal components on a data stream for neural network-based PCA. The presented results demonstrated that the adaptation process is fast and robust. To the best knowledge of the authors, continuously adapting the dimensionality has been rarely applied to neural network-based PCA [7, 46]. Nevertheless, this topic is more frequently mentioned in the field of incremental PCA [8, 9]. Therefore, the proposed method is benchmarked both against the directly related neural network-based method [7] and against an incremental approach [8].

#### Comparison to incremental PCA

The following benchmark is performed on the incremental PCA [8]. The method is in the following benchmarked on all data sets (Table 1), using the cumulative percentage of total variance stopping rule, as suggested by [8]. The results are shown in Table 6, with the same presentation as in all benchmarks before, and can be therefore directly compared.

**Table 6:** Results of the incremental PCA [8] on the cumulative energy stopping rule: *θ*_1_ = 0.7, *θ*_2_ = 0.8, *θ*_3_ = 0.9, *θ*_4_ = 0.99.

	Data set	*μ* ± *s*			
		*θ*_1_	*θ*_2_	*θ*_3_	*θ*_4_
a)	Cameraman_25%_	64.0±0.0	64.0±0.0	64.0±0.0	64.0±0.0
	Cameraman_50%_	1.±0.	1.±0.	5.3±3.4	23.8±7.1
	Cameraman_75%_	1.0±0.0	1.0±0.0	1.0±0.0	3.5±2.5
	Cameraman_100%_	1.0±0.0	1.0±0.0	58.8±1.3	53.8±2.1
	Offline PCA	1	1	4	30
b)	Circle_25%_	1.4±2.0	1.7±2.5	4.4±11.6	7.8±1.6
	Circle_50%_	1.0±0.0	1.0±0.0	1.1±0.3	1.4±1.6
	Circle_75%_	1.0±0.0	1.0±0.0	1.1±0.3	1.2±0.7
	Circle_100%_	1.0±0.0	1.0±0.0	5.7±1.0	2.2±7.6
	Offline PCA	1	1	2	23
c)	PHM08_25%_	1.0±0.0	1.0±0.0	8.0±1.6	11.6±5.5
	PHM08_50%_	1.0±0.0	1.0±0.0	17.1±10.1	23.6±3.2
	PHM08_75%_	1.0±0.0	1.0±0.0	2.0±0.1	23.6±3.2
	PHM08_100%_	1.0±0.0	1.0±0.0	1.9±0.1	7.8±8.2
	Offline PCA	1	1	2	2
d)	CareerCon19_25%_	2.3±0.6	3.4±2.5	5.4±4.0	4.1±3.9
	CareerCon19_50%_	2.1±1.0	3.4±3.1	5.1±4.2	3.7±3.9
	CareerCon19_75%_	1.8±0.5	2.0±1.1	3.7±3.3	3.2±3.6
	CareerCon19_100%_	1.7±1.0	1.7±1.3	1.9±0.9	2.5±2.9
	Offline PCA	2	3	3	5
e)	Waveform_25%_	19.8±0.3	26.2±0.4	19.8±5.7	13.6±5.5
	Waveform_50%_	19.0±0.0	25.8±0.7	15.4±6.9	9.1±5.2
	Waveform_75%_	20.5±0.5	26.7±1.3	14.3±7.5	7.3±5.2
	Waveform_100%_	20.3±0.9	25.8±1.0	13.3±7.7	6.6±5.2
	Offline PCA	18	25	33	40
f)	Waveform2_25%_	6.0±0.2	10.6±2.4	3.0±0.8	3.4±1.3
	Waveform2_50%_	6.0±0.0	7.7±2.9	2.7±0.6	2.8±0.8
	Waveform2_75%_	6.0±0.2	7.4±3.5	2.7±0.6	2.7±0.8
	Waveform2_100%_	6.0±0.0	6.9±3.3	2.7±0.6	2.6±0.7
	Offline PCA	6	11	16	21

**Fig 9:**
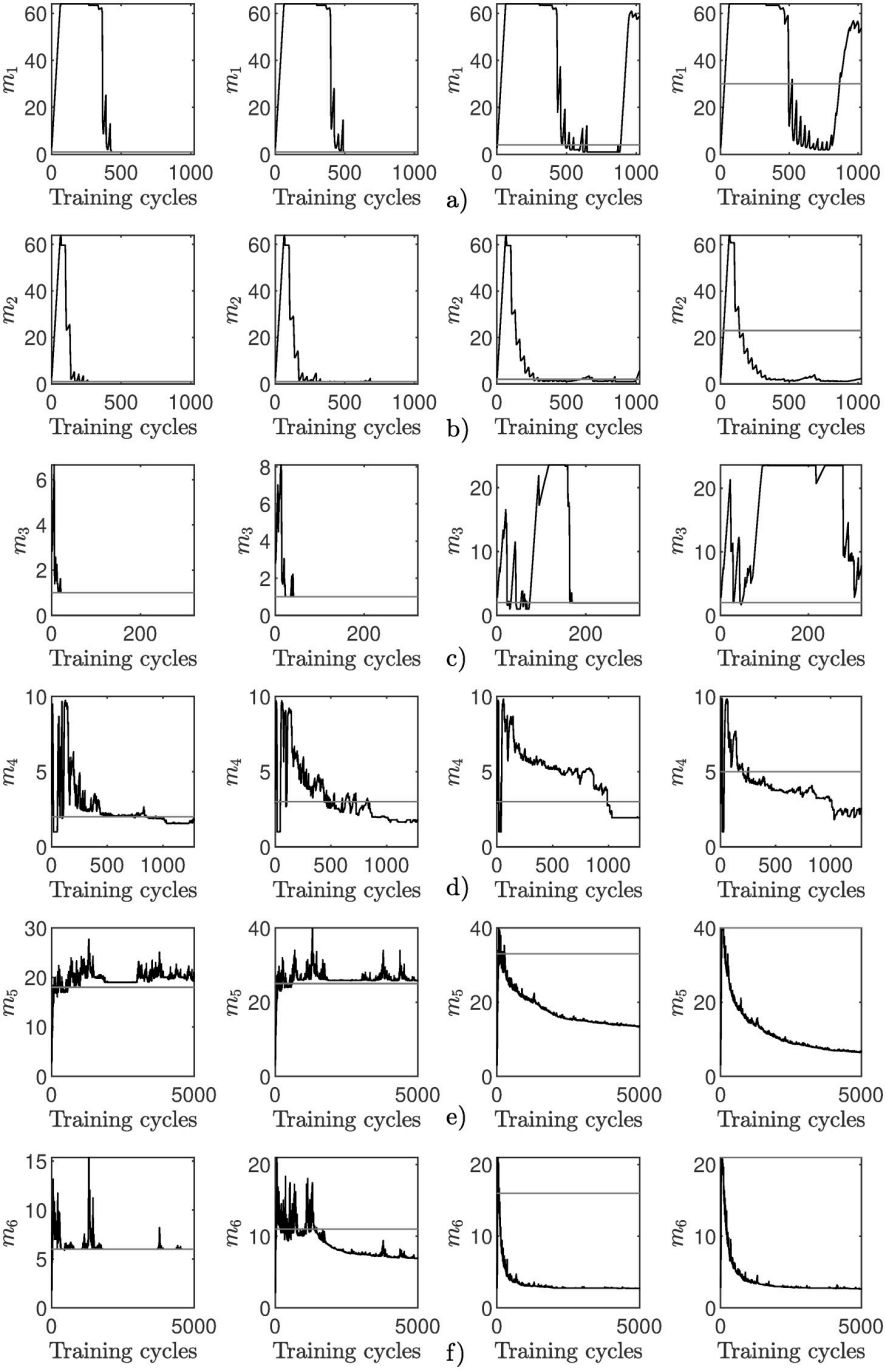
Incremental PCA [8] on the cumulative percentage of total variance stopping rule. The first plot in each row has a proportion of *θ*_1_ = 0.70, the left-middle plot of *θ*_2_ = 0.80, the right-middle plot of *θ*_3_ = 0.90 and the right plot of *θ*_4_ = 0.99: (a-f) shows the results obtained on each data set 1 corresponding to Table 6.

For low percentages of the total variance, such as *θ* = {0.7, 0.8}, the incremental PCA method achieves highly accurate and competitive results. However, on higher thresholds, the accuracy suffers and the correct dimensionality is rarely achieved. In addition, the adjustment process (Fig 9) shows that the method is highly sensitive in the early phase of learning. This can be explained by the exponential decaying term $\frac{N}{(N+1{)}^{2}}$ in the learning rule (10), leading to an over-sensitive behavior in the initial learning phase. In comparison to the method proposed in this work, incremental PCA achieves comparable results on lower thresholds but not on higher ones.

#### Comparison to neural network-based PCA

The approach [7] consists of three main steps that are described as follows:

1. Initialization: Set the dimensionality to *m* = 1, the eigenvalue λ_1_ to a random value and the corresponding eigenvectors to a random orthonormal system.
2. Training: Present a data point **x** and update the parameter set.
3. Dimensionality adjustment: Check if the stopping rule condition is fulfilled or not. Either add *m* = *m* + 1 or remove *m* = *m* − 1 one dimension.
4. Repeat: Continue with step 2.

This approach optimizes the dimensionality by adding or removing one dimension whenever a new data point is collected. The approach is compared with the novel approach presented in this work. For this purpose, the competing approach [7] is tested on some of the data sets (Table 1). The benchmark is limited to a slightly modified version of the cumulative percentage of total variance stopping rule (14), which was used in [7]. If the predefined cumulative percentage of the total variance is not described with the current number of principal components, one dimension is added and vice versa.

**Fig 10:**
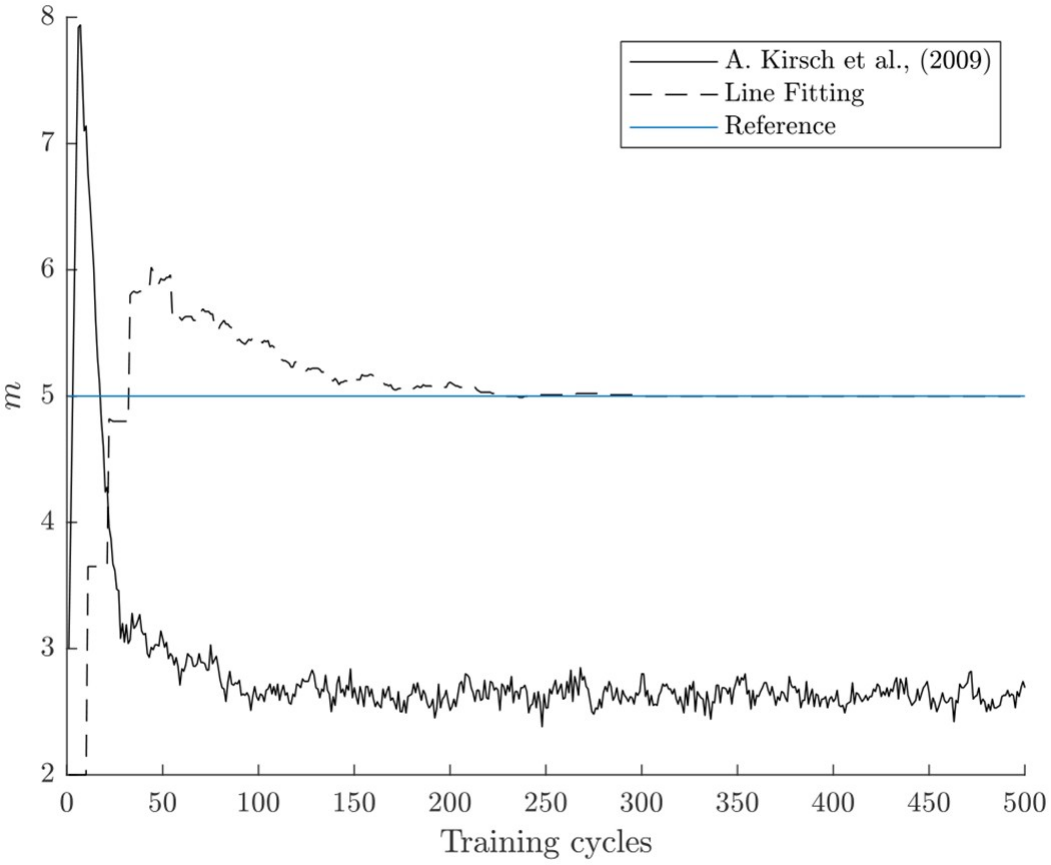
Dimensionality adjustment process on the CareerCon19 [51] data set with different approaches and *θ* = 0.99. The horizontal line is a reference dimensionality calculated with an offline PCA. The solid line is the dimensionality adjustment process with the novel approach presented in this work, while the dashed line represents [7]. Each data point in the other two line is the mean of 100 repetitions.

The achieved results are visualized in Fig 10. The reference dimensionality *m* = 5 is calculated with an offline PCA. The competing approach immediately overshoots and then drops below the optimal dimensionality. For the rest of the training process the approach is unable to achieve the correct dimensionality. In comparison, the novel approach presented in this work adapts quickly towards the optimal dimensionality and keeps it.

The results showcased are supported by a larger experimental study that is too extensive to be shown in this work. In conclusion, the novel approach presented in this work is superior to the competing approach [7] in many ways. While the competing approach is only capable of adding or removing one dimension, the novel algorithm is able to add or remove any number of dimensions per training cycle. Additionally, the adaptation process is faster and more robust against initial overshoots. Most importantly, the correct final dimensionality is reached and held more reliably.

### Investigation on the computational complexity

Applied machine learning is often limited by available memory and resources. Hence, it is necessary to consider the computational complexity. The computational complexity is used to describe to which function the computation time is asymptotically proportional. For PCA algorithm, it is always considered in connection with an input dimensionality *n* and the amount of considered data points *N*. In the following, the complexity of offline PCA, neural network-based PCA, the proposed extension, and incremental PCA are compared to each other.

In offline PCA, the eigenvalues **Λ** and eigenvectors **W** are obtained with the eigenvalue decomposition of the covariance matrix. As preparation, the data $\text{X}\in {\mathbb{R}}^{n\times N}$ are first centered. In the next step, the centered data are multiplied with its transpose to obtain the covariance matrix. This matrix multiplication is computationally expensive ((*n* × *N*) and (*N* × *n*)) leading to a computational complexity of O(nNmin(n,N)) for the covariance estimation. Decomposing the eigenvalues of the covariance matrix costs in a worst case scenario with a *n* × *n* matrix $O\left(n^{3}\right)$. Thus, the total complexity for offline PCA is $O\left(nN\text{min}(n,N)+n^{3}\right)$, which can be further reduced [17].

However, the classical PCA described above is performed on all training data simultaneously, which requires to have all data in advance. This is not the case in an online case, where data are processed sequentially. Therefore, the eigenvalues and eigenvectors have to be updated whenever a new data point is presented. Neural network-based PCA is an approach to continuously update the eigendirections after each data point representation [41]. Given a data point dimensionality *n* and a reduced dimensionality *m*, then the complexity is given with O(nm) [17]. A robust extension (RRLSA) [32] exhibits faster convergence and higher stability, but the complexity increases to O(nm^2^). Integrating a Gram-Schmidt orthonormalization into the RRLSA maintains the same complexity O(nm^2^) [34], despite more elementary operations are required. The new method for adaptive dimensionality adjustment presented in this work performs a linear regression based on the reduced set of *m* eigenvalues. Generally, a linear regression has a computational complexity of O(mp^2^ + p^3)^ for a *m* × *p* matrix, due to the matrix multiplication and inversion. Since the linear regression used here only relies on a vector containing the first *m* eigenvalues, the computational complexity reduces to O(m). Despite, is O(nm^2^) always growing faster, so that the impact of the linear regression is omitted. Incremental PCA, as presented in [8, 40], is capable of incrementally updating a eigenspace model with a complexity of O(nm^2^). Thus, overall both incremental and neural network-based PCA offer computationally efficient ways of updating the eigendirections.

### Investigation of a double-logarithmic approach

The results achieved in the previous sections demonstrated it is possible to accurately predict eigenvalues with a linear regression model in the logarithmic scale, if combined with a hierarchical online PCA. While the results in section showed the benefit of the proposed algorithm compared to a competing approach, it is the goal of this section to clarify if operating in the double-logarithmic scale is a viable alternative.

The idea to observe the eigenvalue in a double-logarithmic scale was discussed for offline PCA [47]. The double-logarithmic transformation faces the same problem as the single-logarithmic transformation in offline PCA because the error between real and approximated eigenvalues is too large to make a precise approximation. Due to the continuous eigenvalue training this problem does not exist in hierarchical online PCA.

Using a double-logarithmic scale, the values of the remaining *n* − *m* eigenvalues are determined via

$${\tilde{\lambda }}_{i}^{*}=\alpha \;\text{log}(i)+\beta ,$$ (24)

$$\lambda_{i}^{*}=\text{exp}({\tilde{\lambda }}_{i}^{*})$$ (25)

with *i* ∈ {*m* + 1, …, *n*}. Instead of testing all stopping rules, the comparison is limited to the cumulative percentage of total variance stopping rule because this method achieved the best results. The results are presented in Table 8.

In the low-dimensional area with *θ* = [0.7, 0.8], the double-logarithmic approach is in most cases able to predict the correct final dimensionality with slightly higher standard deviations *s* compared to the single-logarithmic approach. For the image data sets, the approach cannot correctly estimate the dimensionality for *θ* = 0.99. Additionally, the standard deviation in the upper area of *θ* ≥ 0.9 is consistently larger than in the single-logarithmic approach.

This additional experiment was carried out to test if using the double-logarithmic scale to estimate the eigenvalues is superior to using the single-logarithmic scale. However, the results demonstrate that this method yields worse results on the given data sets and applied stopping rule.

## Conclusion

Driven by the technological developments in all areas of our live and the explosion in available heterogeneous data, the further advancement of dimensionality reduction methods is highly relevant. The method proposed in this work enhances neural network-based PCA by an algorithm to accurately determine the optimal number of meaningful principal components on data streams.

Therefore, neural network-based PCA was extended by an algorithm that is capable of adjusting the dimensionality in large step size at every timestep. The algorithm takes advantage of natural characteristics of neural network-based PCA.

The approach for adaptive dimensionality adjustment in neural network-based PCA was tested on a variety of data sets. The criteria to rate the adjustment quality were firstly the accuracy (Tables 2–8) and secondly the convergence speed (Figs 5–8). Different stopping rules were explored: (1) The eigenvalue approximation in combination with the eigenvalue-one criterion was not able to find the correct dimensionality when the data set is augmented with many noise dimensions. (2) In contrast, combining the eigenvalue estimation with the eigenvalue-average stopping rule through replacing the fixed threshold with a moving threshold, already yielded better results. The adaptation process was faster and the remaining standard deviation was lower. (3) For the percentage of total variance criterion, an extra factor *η* was introduced, which defines the minimal represented variance to be preserved by the principal components. The algorithm showed its strength by accurately predicting *n* − *m* eigenvalues. In this way, the quality of the results could be further increased. (4) The cumulative percentage of total variance criterion correctly calculated the dimensionality in all but one combination of data set and parameter *θ*. All in all, the proposed algorithm for eigenvalue estimation and adaptive dimensionality adjustment proved to be applicable to online PCA and correctly approximated the final dimensionality long before all data points were presented.

**Table 7:** Comparison of algorithmic complexity regarding computation time and memory consumption for each data point presentation.

Method	Memory storage	Computational time
Batch (EVD)	O(Nn)	O(Nn⋅min(N,n))
NN-PCA	O(nm)	O(nm)
NN-PCA (orth.)	O(nm)	O(nm^2^)
RRLSA	O(nm)	O(nm^2^)
RRLSA (orth.)	O(nm)	O(nm^2^)
RRLSA (orth.) with Dim. adjustment	O(nm)	O(nm^2^)
Incremental PCA	O(nm)	O(nm^2^)

**Table 8:** Cumulative percentage of total variance with double-logarithmic eigenvalue approximation, *θ*_1_ = 0.7, *θ*_2_ = 0.8, *θ*_3_ = 0.9, *θ*_4_ = 0.99.

	Data set	*μ* ± s			
		*θ*_1_	*θ*_2_	*θ*_3_	*θ*_4_
a)	Cameraman_25%_	1.1±0.3	1.7±0.9	6.1±4.1	16.4±7.1
	Cameraman_50%_	1.0±0,0	1.4±0.5	5.9±5.1	9.6±9.6
	Cameraman_75%_	1.0±0.0	1.2±0.4	5.7±6.3	8.7±9.6
	Cameraman_100%_	1.0±0.0	1.2±0.4	6.0±6.9	13.5±11.0
	Offline PCA	1	1	4	30
b)	Circle_25%_	1.1±0.4	1.3±0.6	3.4±2.5	11.8±6.5
	Circle_50%_	1.0±0.0	1.1±0.3	3.3±3.0	11.0±9.1
	Circle_75%_	1.0±0.0	1.0±0.1	4.2±6.7	9.0±9.3
	Circle_100%_	1.0±0.0	1.0±0.0	4.3±8.7	9.8±8.7
	Offline PCA	1	1	2	23
c)	PHM08_25%_	1.0±0.1	1.1±0.3	1.8±0.5	2.5±0.8
	PHM08_50%_	1.0±0.0	1.1±0.0	1.9±0.3	2.0±0.4
	PHM08_75%_	1.0±0.0	1.0±0.0	1.9±0.3	1.9±0.3
	PHM08_100%_	1.0±0.0	1.0±0.0	2.0±0.2	1.9±0.3
	Offline PCA	1	1	2	2
d)	CareerCon19_25%_	2.4±0.6	2.9±0.8	3.6±0.8	4.9±0.4
	CareerCon19_50%_	2.2±0.6	2.8±0.7	3.2±0.4	4.9±0.4
	CareerCon19_75%_	2.1±0.3	2.9±0.5	3.1±0.3	5.0±0.0
	CareerCon19_100%_	2.0±0.2	2.9±0.4	3.0±0.2	5.0±0.0
	Offline PCA	2	3	3	5
e)	Waveform_25%_	17.9±2.1	26.1±2.9	33.2±1.4	39.6±1.9
	Waveform_50%_	18.0±2.0	26.6±2.7	33.1±1.4	39.6±1.9
	Waveform_75%_	18.4±1.9	26.6±2.7	33.0±1.3	39.6±1.9
	Waveform_100%_	18.6±1.9	26.5±2.7	33.0±1.3	39.6±1.9
	Offline PCA	18	25	33	40
f)	Waveform2_25%_	5.8±1.3	10.8±1.2	16.1±0.3	20.9±0.1
	Waveform2_50%_	5.6±1.2	11.0±1.0	16.0±0.2	21.0±0.0
	Waveform2_75%_	5.7±1.2	11.1±0.9	16.0±0.2	21.0±0.0
	Waveform2_100%_	5.8±1.2	11.1±0.9	16.0±0.1	21.0±0.0
	Offline PCA	6	11	16	21

The approach was in the following benchmarked against existing neural network-based and incremental PCA algorithms for adaptive dimensionality adjustment, both methods being limited to an increment of one dimension per data point. The methods were compared on the cumulative percentage of total variance stopping rule. However, the newly proposed method achieved considerably better results in terms of accuracy and speed.

In addition, it was tested if the results could be further improved by fitting the linear regression model, which operates at the core of the proposed method, in the double logarithmic scale. This method was tested in combination with the cumulative percentage of total variance stopping rule. The results in the double-logarithmic scale were less stable, therefore the single-logarithmic scale is preferable.

## List of symbols

**c**: PCA center
**C**: covariance matrix
*i*: eigenvalue index
*K*: total number of training steps
*m*: number of eigenvectors
*n*: dimensionality of data space
*N*: number of samples in incremental PCA
*U*: supplemented set of trained and estimated eigenvalues
*V*: set of trained eigenvalues
**W**: matrix of estimated eigenvectors
**w**_*i*_: i^th^ eigenvector estimate
$\tilde{U}$
supplemented set of trained and estimated log-eigenvalues
$\tilde{V}$
set of trained log-eigenvalues
${\tilde{V}}^{*}$
set of estimated log-eigenvalues
**x**: vector drawn from data space
**X**: set with training data
**y**: neuron activation
*α*: regression slope
*β*: regression offset
*δ*: adaptive learning rate
Γ: initial training parameter
*η*: proportion factor
*θ*: complexity factor
*κ*: current training step
λ: trained eigenvalue
λ_total_: total variance
$\tilde{\lambda }$
trained log-eigenvalue
${\tilde{\lambda }}^{*}$
estimated log-eigenvalue
**Λ**: diagonal matrix of eigenvalues
*σ*^2^: residual variance
*φ*: activation function of a neuron
*μ*: low-pass filter parameter
*ψ*: adaptive learning rate
*ξ*: distance between data and center

## Supporting information

S1 Data Availability of data, code and material. (PDF)

S1 Appendix Learning rate control. (PDF)
