# Supplementary material for: Adaptive dimensionality reduction for neural network-based online principal component analysis
Source: PLoS One. 2021 Mar 30;16(3):e0248896. doi: 10.1371/journal.pone.0248896 (PMC8009402; doi:10.1371/journal.pone.0248896)
Supplement: S1 Data — (PDF) [file pone.0248896.s001.pdf]

S1 Data. **Availability of data, code and material.** The data sets that support the findings of this study are freely available from [49–52]. The code generated and analysed in this work is available in the "Adaptive-Dimensionality-Adjustment" repository,

"https://github.com/NicoMigenda/Adaptive-Dimensionality-Adjustment". In the same repository, a MATLAB implementation of the incremental PCA [8] that is used for the benchmark can be found.
