# Supplementary material for: Adaptive dimensionality reduction for neural network-based online principal component analysis
Source: PLoS One. 2021 Mar 30;16(3):e0248896. doi: 10.1371/journal.pone.0248896 (PMC8009402; doi:10.1371/journal.pone.0248896)
Supplement: S1 Appendix — (PDF) [file pone.0248896.s002.pdf]

S1 Appendix. Learning rate control. Choosing a proper learning rate in neural network-based PCA is crucial for convergence. Most learning rates are declining exponentially or require hyper-parameter tuning [17]. The adaptive learning rate used for the results in this paper is based on a variance matching between the eigenvalues $\mathbf{\Lambda}$ and the neuron output $\mathbf{y} = \mathbf{W}^T(\mathbf{x} - \mathbf{c})$. This procedure is performed element-wise for each dimension m. When the neuron output and the eigenvalue are equal with $\frac{y_M^2}{\lambda_M} = 1 \,|\, M = \{1, 2, \ldots, m\}$, a good fit is considered. The new fit is calculated by adding up the previous and current fit with a low-pass filter. In this way, a matching vector

$$\hat{\boldsymbol{\delta}} = (1 - \mu)\hat{\boldsymbol{\delta}} + \mu \left(\mathbf{y} \odot \mathbf{y} \oslash \mathbf{\Lambda}^{-1}\right) \tag{26}$$

is calculated where the Hadamard product $\odot$ and division $\oslash$ are element-wise operations. To achieve a learning rate in the interval $[0, 1]$, the matching vector $\hat{\boldsymbol{\delta}}$ has to be normalized and converted to a scalar. The normalized matching parameter in the range $[0, 1]$, in the following used as the learning rate, is calculated by

$$\delta = \frac{\sum_{i=1}^{m} \min\left(|\log_{10}\left(\hat{\delta}_i\right)|, 1.0\right)}{m} \tag{27}$$

with δ being the learning rate used in neural network-based PCA learning rules (1)-(3). For each dimension m of the matching vector $\hat{\delta}_i$, in (27), the logarithm to the base of 10 is taken. For a perfectly matching dimension ($\delta = 1$) the logarithm and therefore the entire equation (27) will be 0. In that case, no further adjustment of the PCA parameters (1)-(3) is performed. In other cases the absolute value is taken to force the learning rate into the positive scale. In order to prevent values greater 1, the value of each element is limited to 1. Lastly, the learning rate δ is averaged over all dimensions.
